# Multi-omics integration reveals vaccine-specific molecular signatures and temporal dynamics of COVID-19 immune responses

**DOI:** 10.3389/fimmu.2026.1815543

**Published:** 2026-10-02

**Authors:** Anne H. Klein, David J. Beale, Thao V. Nguyen, Jian-Wei Liu, Petrus Jansen van Vuren, Nagendrakumar B. Singanallur, Carol Lee, Andrew C. Boomer, Jennifer A. Juno, Stephen J. Kent, Adam K. Wheatley, Gough G. Au, Seshadri S. Vasan, Laurence Wilson, Alexander J. McAuley

**Affiliations:** 1 Australian eHealth Research Centre, Commonwealth Scientific and Industrial Research Organisation (CSIRO), Herston, QLD, Australia; 2 Environment, Commonwealth Scientific and Industrial Research Organisation (CSIRO), Ecosciences Precinct, Dutton Park, QLD, Australia; 3 Environment, Commonwealth Scientific and Industrial Research Organisation (CSIRO), Black Mountain, Clunies Ross Street, Acton, ACT, Australia; 4 Australian Centre for Disease Preparedness (ACDP), Commonwealth Scientific and Industrial Research Organisation (CSIRO), East Geelong, VIC, Australia; 5 Department of Microbiology and Immunology, University of Melbourne at The Peter Doherty Institute for Infection and Immunity, Melbourne, VIC, Australia; 6 Edith Cowan University, School of Medical and Health Sciences, Joondalup, WA, Australia; 7 Department of Biomedical Sciences, Macquarie University, Sydney, NSW, Australia

**Keywords:** COVID-19 vaccination, multi-omics, proteomics, lipidomics, metabolomics, Pfizer-BioNTech, Moderna, AstraZeneca

## Abstract

Vaccination elicits systemic immune responses that extend beyond antibody and lymphocyte activation, engaging coordinated metabolic and lipid pathways that modulate immune function. Nevertheless, how systemic molecular responses differ across COVID-19 vaccine platforms, including closely related mRNA platforms, remains poorly understood. Here, we performed integrated plasma proteomic, lipidomic, and metabolomic profiling of 53 SARS-CoV-2–naïve adults vaccinated with Pfizer-BioNTech, Moderna, or AstraZeneca COVID-19 vaccines. Samples were analysed across multiple timepoints using differential analysis and DIABLO-based multi-omics integration. Our findings reveal distinct platform-associated multi-omic molecular signatures not captured by unsupervised analyses alone. Pfizer-BioNTech vaccination was associated with widespread increases in immunoglobulin and complement associated proteins, together with broad decreases in lipid abundance and bidirectional metabolite changes. In contrast, the Moderna vaccination was associated with predominantly increased lipid abundance and bidirectional metabolite changes, with minimal univariate proteomic changes. AstraZeneca induced subtler yet coherent cross-omic shifts, detectable only through integrative modelling approaches. Despite these platform-associated differences, Pfizer and Moderna shared concordant changes in a subset of metabolites, including amino-acid and TCA-cycle-associated features, within divergent lipidomic contexts. These findings demonstrate that even closely related mRNA vaccine platforms are associated with distinct systemic molecular response patterns. This study establishes integration of multi-omics (as opposed to separate single-omic analyses) as a powerful framework for characterising vaccine-associated molecular responses, identifying candidate platform-specific molecular markers, and providing candidates for future molecular stratification studies.

## Introduction

Popular COVID-19 vaccines have encompassed diverse technological platforms, including mRNA-based formulations (e.g. Pfizer-BioNTech, Moderna) and viral vector systems (e.g. AstraZeneca), each employing distinct mechanisms to present SARS-CoV-2 antigens to the immune system (1–4). In addition to their different delivery modalities, these platforms vary in their adjuvant properties, cellular uptake mechanisms, potentially leading to divergent molecular and immunological outcomes (5, 6). However, current vaccine monitoring approaches often treat immune responses as largely uniform across platforms, overlooking potentially important differences in the breadth, kinetics, and quality of their associated molecular responses (7, 8). The immune response to vaccination involves more than antibody production and T-cell activation. Emerging evidence from the field of immunometabolism reveals that immune cells undergo profound metabolic reprogramming to support their effector functions (9–11), whereas lipid mediators coordinate inflammatory resolution and tissue homeostasis (12–14). Simultaneously, complement cascades, acute-phase responses (15, 16), and cytoskeletal reorganisation (17) create a complex molecular symphony that determines the magnitude and quality of vaccine-induced protection. Understanding and interpreting this complexity requires moving beyond single-analyte measurements to systems-level approaches that capture the full spectrum of host responses. Multi-omics profiling offers unprecedented opportunities to decode the molecular basis of vaccine response (18–22). By simultaneously measuring proteins, lipids, and metabolites, we can begin to understand how different biological layers coordinate to generate immunity (23–27). However, traditional approaches that analyse each omics dataset independently may miss critical interlayer relationships and fail to identify the most biologically relevant signatures. Integrative analytical frameworks are therefore essential to uncover the coordinated molecular patterns that distinguish vaccine-specific responses and to identify clinically actionable biomarkers. The clinical implications of understanding vaccine-specific molecular signatures extend far beyond basic biology (28, 29). As booster strategies evolve and new variants emerge, the ability to monitor and predict individual vaccine responses could enable personalised vaccination approaches (30, 31). Furthermore, identifying molecular biomarkers of vaccine efficacy could accelerate the development of next-generation vaccines and provide early indicators of protection and adverse responses. Such precision medicine approaches require a fundamental understanding of how systemic molecular responses differ across vaccine platforms. Despite the critical importance of these questions, comprehensive multi-omics studies integrating proteomics, metabolomics, and lipidomics to characterize COVID-19 vaccine responses remain sparse (32–34), and head-to-head comparisons between major vaccine platforms using fully integrated molecular profiling remain limited. The temporal dynamics of these responses, particularly the coordination between different molecular layers over time, remain poorly understood (35). Moreover, the identification of vaccine-associated molecular signatures remains relatively unexplored.

While prior systems vaccinology studies have primarily employed transcriptomic profiling to identify early immune signatures predictive of vaccine efficacy, such approaches often overlook downstream molecular events that occur beyond gene expression (19, 36). These transcriptome-centric studies provide valuable insights into early innate immune activation, but do not capture post-transcriptional, metabolic, or lipid-mediated regulatory processes that shape immune responses. In contrast, our study employs an integrated multi-omics approach, combining proteomics, lipidomics, and metabolomics, to characterise the systemic molecular responses associated with three major COVID-19 vaccines. By profiling plasma from individuals vaccinated with Pfizer-BioNTech, Moderna, or AstraZeneca across multiple timepoints, we aim to: (1) characterise platform-specific molecular signatures, (2) identify cross-omic associations among molecular features, (3) understand temporal dynamics of vaccine-induced programs, and (4) identify candidate molecular markers for future validation as potential biomarkers of vaccine-associated molecular signatures.

## Methods

### Study design and cohort

Human plasma samples were obtained from 53 healthy individuals with no prior history of SARS-CoV-2 infection who received BNT162b2 (Pfizer-BioNTech; n=22), mRNA-1273 (Moderna; n=24), and ChAdOx1 nCoV-19 (Oxford–AstraZeneca; n=7), as previously described (37). The study protocols were approved by institutional ethics committees of the University of Melbourne, Australia [University of Melbourne Central Human Research Ethics Committee (2021-21198-15398-3)] and the CSIRO (CSIRO Human Research Ethics Committee ID 2021_123_RR and 2025_036_LR). Because participants contributed samples at multiple timepoints, the analytical dataset comprised 137 plasma samples in total: Pfizer 75, Moderna 48, AstraZeneca 14. All platforms were sampled at Baseline and 2 weeks after the 2nd dose (2wk-2nd). Extended longitudinal sampling was performed only for Pfizer-BioNTech recipients, with additional collections at 6 months after the 2nd dose (6mo-2nd) and 2 weeks after the 3rd dose (2wk-3rd). Not all Pfizer participants contributed at every longitudinal timepoint, hence the total of 75 Pfizer samples. Participant metadata included sex, year of birth, and vaccine received, with age calculated from year of birth. A detailed breakdown of participant demographics and sample availability by vaccine platform and timepoint is provided in **Table 1**.

**Table 1:** Participant demographics and sample distribution by vaccine platform and timepoint.

Vaccine	Participants (n)	Age, mean ± SD (years)	Age range	Female, n (%)	Male, n (%)	Baseline samples	2wk-2nd samples	6mo-2nd samples	2wk-3rd samples
**Pfizer-BioNTech**	22	36.27 ± 9.98	25–57	14 (63.6%)	8 (36.4%)	22	22	16	15
**Moderna**	24	46.33 ± 16.05	27–84	11 (45.8%)	13 (54.2%)	24	24	Not collected	Not collected
**AstraZeneca**	7	49.86 ± 16.40	22–65	4 (57.1%)	3 (42.9%)	7	7	Not collected	Not collected
**Total**	53	42.62 ± 14.69	22–84	29 (54.7%)	24 (45.3%)	53	53	16	15

Boldface indicates vaccine-group labels and the Total row only.

### Plasma omics profiling: proteomics, lipidomics, and metabolomics

Metabolites, lipids and proteins were extracted from 100 μL of human plasma using a one-pot extraction method previously described in (38). Briefly, plasma was quenched with 450 μL of ice-cold (−20 °C) methanol:ethanol (1:1, v/v; LiChrosolv^®^, Merck, Darmstadt, Germany) spiked with 100 ppb Phenylalanine (1-^13^C) (Cambridge Isotope Laboratories, Andover, MA, USA), and vortexed for 2 min. The samples were centrifuged (Centrifuge 5430R, Eppendorf, Hamburg, Germany) at 14,000 rcf at 4 °C for 5 min to pellet the protein which was set aside for proteome measurement. The supernatant was transferred and filtered using a positive pressure manifold (Agilent PPM48 Processor, Agilent Technologies, Santa Clara, California, USA) with Captiva EMR cartridges (40 mg, 1 mL; Agilent Technologies, Mulgrave, VIC, Australia) to separate the lipid and metabolite fraction. The EMR cartridge was washed with two 200 µL aliquots of RO water:methanol:ethanol (2:1:1, v/v), with the flow-through supernatant (comprising polar metabolites) and cartridge washes collected in LC-MS grade high recovery vials and dried under a stream of high purity (RATEK-Evaporation manifold, AdelLab Scientific, Thebarton, South Australia). The non-polar lipid fraction retained on the EMR cartridge was then eluted with two 500 µL aliquots of dichloromethane:methanol (1:2, v/v; LiChrosolv^®^, Merck, Darmstadt, Germany) into a second LC-MS grade high recovery vial and dried under a stream of nitrogen. The polar metabolite fraction was reconstituted with 100 μL MilliQ:methanol (8:2, *v*/v) spiked with 100 ppb of succinic acid (1,4-^13^C_2_) (Cambridge Isotope Laboratories, Andover, MA, USA) and the lipid fraction was reconstituted in 100 μL butanol:Methanol (1:1, v/v). The analytical work list comprised procedural blanks (n=7) and pooled biological quality control (PBQC) samples (n=7) that were analysed throughout the sequence. The residual relative standard deviation (RSD%) of the internal standards were 4.89% (l-Phenylalanine, 1-^13^C), and 1.95% (Succinic Acid, 1,4-^13^C _2_). Total protein concentration was estimated using a Bradford assay. Protein pellets were resuspended in 2000 µL of 8M Urea, 20mM ammonium bicarbonate. Protein concentration was determined using a Bradford assay kit according to the manufacturer (Bio-Rad). An aliquot of proteins (3 µg) was reduced and alkylated. Proteins were then digested with trypsin by incubation at 37°C for 3–4 hours. The digestion was stopped with 0.1% of formic acid and filtered with a 0.22µm filter.

Central carbon metabolism metabolites (CCM) were measured on an Agilent Infinity Flex II UHPLC coupled to an Agilent 6470 Triple Mass Spectrometer (QqQ-MS) following (39); untargeted lipids and metabolites were analysed on an Agilent Infinity Flex II UHPLC coupled to an Agilent 6546 Quadrupole Time-of-Flight Mass Spectrometer (QToF-MS) following (38). Untargeted QTOF data were acquired in both MS and AutoMS/MS modes, with fragmentation spectra collected at collision energies of 0, 10, 20, and 40 eV for metabolite annotation. The trypsin digested protein samples were analysed using an Ultimate™ 3000 RSLC nano LC system (Thermo Scientific) following previous methods (40–42).

Central carbon metabolism metabolites were processed using MassHunter Quantitative software (Version 10.1, Agilent Technologies, Santa Clara, CA, USA), normalized to IS. Central carbon metabolites quantified using the targeted dMRM assay were confirmed against authentic analytical standards and were classified as Schymanski Level 1 (confirmed structure). Untargeted metabolite and lipid data were processed using MassHunter Explorer software (Version 2.0, Agilent Technologies, Santa Clara, CA, USA), normalized to IS, and putatively identified against the Agilent METLIN Metabolite and Lipid PCDLs (G6825-90008, Agilent Technologies, Santa Clara, CA, USA) and a curated in-house PCDL based on retention time (± 0.15 min), precursor ions, tandem mass spectrometry (MS/MS) spectra and a library threshold score of more than 80%. Annotation confidence for untargeted metabolites was assigned according to the framework of Schymanski et al. (2014) (1). Compounds supported by accurate mass, retention time, and diagnostic MS/MS fragmentation spectra, but lacking confirmation with an authentic analytical standard, were classified as Level 2 (probable structure). Features for which multiple plausible structural candidates remained were classified as Level 3 (tentative candidate), features assigned only a molecular formula were classified as Level 4, and features characterised solely by exact mass were classified as Level 5. For the acquired lipid data, auto MS/MS data on pooled PBQC samples were obtained at collisions of 20 eV and 35 eV. The acquired MS/MS lipid data were further analysed using the Agilent Lipid Annotator tool (V1.0; Santa Clara, CA, USA) which assigned isomeric structures based on MS/MS fragmentation patterns. Lipid species supported by accurate mass, retention time, and diagnostic fragmentation patterns, but without confirmation against authentic analytical standards, were reported as Schymanski Level 2 annotations. Annotated lipids were then curated into a PCDL, which was used to identify lipids within the remaining analysed samples with retention time thresholds (± 0.15 min), MS/MS spectra, and a library threshold score of more than 80%. The curated lipid PCDL incorporated accurate mass, retention time, and MS/MS fragmentation information together with screening against the Agilent METLIN Lipid PCDL. Protein Discoverer 2.2 (Thermo Scientific) and Sequest HT search engine were used to identify peptides/proteins and quantify relative abundance of proteins. The spectrum data was searched against the UniProt *Homo sapiens* database (Proteome ID: UP000005640, 20,311 sequences). Precursor mass tolerance was set to 10ppm and product ions were searched at 0.6 Da. Three missed tryptic cleavages were allowed. Modification included Oxidation (+ 15.995Da), Deamidation (+ 0.984 Da), Amidation (- 0.984Da), and Propionamidation (+ 71.037 Da). Peptide spectral match was validated using the Percolator algorithm, based on q-values and 1% False Discovery Rate (FDR). Relative abundance is calculated from precursor abundance intensity and is normalized from total peptide amount.

### Omics data preprocessing and quality control

For each available plasma sample, untargeted proteomics, metabolomics, and lipidomics profiling and targeted central carbon metabolism metabolites was performed in parallel. Systematic quality control was applied to all proteomic, lipidomic, and metabolomic datasets. Non-numeric entries and missing value indicators were converted to NA across all platforms. For lipidomic data, vendor-encoded default values (intensity = 1) were specifically converted to missing values. Platform-specific intensity thresholds were applied to metabolomics data to distinguish biological signal from technical noise: values <300 for the targeted CCM platform and <1,600 for untargeted QToF platforms were treated as non-detections. Features were excluded if missing values exceeded 40% overall for proteomics due to inherent peptide detection limits, dynamic range limitations, and stochastic sampling of low-abundance proteins; 35% for lipidomics due to moderate technical variability from lipid extraction efficiency and differential ionisation; and 30% for metabolomics reflecting the higher sensitivity and more comprehensive coverage of small molecule detection platforms. Group-specific thresholds of 60% (proteomics/lipidomics) or 50% (metabolomics) were applied within pre or post-vaccination groups to ensure sufficient observations for valid fold-change estimation. To evaluate the consistency of baseline molecular profiles across vaccine platforms, we performed a cross-platform comparison of pre-vaccination (baseline) samples after applying platform-specific normalisation. This assessment included PCA, distributional profiling, and differential testing across vaccine groups at baseline. The goal was to rule out systemic artefacts, technical biases, or group-wise pre-vaccination differences that could confound downstream analyses. Potential technical or batch-related structure was assessed using distributional QC, sample–sample correlation matrices, and PCA, as detailed in the **Supplementary Methods**.

### Data preprocessing and normalisation benchmarking

We compared six normalisation pipelines (Default, VSN, Quantile Z-score, Log_2_ Autoscale, Loess, Complete), each defined by a fixed imputation–transformation–scaling recipe (**Table 2**). To ensure comparability across pipelines, we first applied a common two-stage, omics-specific feature filter; full thresholds and diagnostics are provided in **Supplementary Method**. Each pipeline was then run on the same filtered matrix for each omics layer. Pipeline performance was assessed by (i) variance captured by PC1–PC3 from a centred/scaled PCA and (ii) a centroid-separation metric (mean Euclidean distance between group centroids in PC space). To respect the unbalanced design, timepoint separation was computed only within timepoints shared across vaccines, and vaccine separation only within timepoints shared across platforms.

**Table 2:** Overview of preprocessing pipelines.

Pipeline	Imputation	Normalisation	Scaling
Default	min/3	Min–max	Pareto
VSN pipeline	min/3	VSN	None
Quantile Z-score	KNN	Quantile	Z-score
Log2 Autoscale	QRILC	log_2_(x+1)	Z-score
Loess pipeline	Mean	Cyclic loess	Z-score
Complete pipeline	min/3	log_10_(x+0.001)	Pareto + Z-score

### Differential expression analysis

Differential expression (DE) was assessed using the limma framework implemented in R (limma package version 3.62.2). For each vaccine platform and omics layer, baseline samples were contrasted with post-vaccination samples. In Pfizer recipients, additional contrasts were examined at 2wk-2nd, 6mo-2nd, and 2wk-3rd. Primary vaccine-specific models included vaccination status together with age and sex covariates, and empirical Bayes moderation was applied to variance estimates across features. To assess the impact of repeated measurements, complementary sensitivity analyses were performed at the shared baseline and 2wk-2nd timepoints using (i) linear mixed-effects models including participant as a random intercept and (ii) participant-level paired change scores. These models additionally included vaccine, age, and sex and were used to assess whether the principal conclusions were robust to explicit modelling of within-participant dependence. For each feature, log_2_ fold change, moderated t statistic, and both raw and adjusted P-values were calculated. Multiple testing was controlled using the Benjamini–Hochberg procedure, and features were considered significant at a false discovery rate of less than 0.05, with additional effect-size thresholds of an absolute log_2_ fold change greater than 0.585 and a moderated t statistic greater than 2. These combined criteria were selected conservatively to require control of multiple testing, a non-trivial magnitude of change, and strong statistical support from the moderated test statistic. To assess the robustness of downstream differential-abundance conclusions to preprocessing choice, all vaccine–omics comparisons were additionally repeated using alternative implemented normalisation pipelines while holding sample inclusion, feature filtering, covariate adjustment, limma modelling, and multiple-testing procedures constant. Robustness was assessed using Spearman correlation of model-derived effect estimates, concordance of effect direction, overlap of FDR-significant features, and Jaccard similarity between significant feature sets.

### Multi-omics integration (DIABLO)

Integrated analyses of proteomic, lipidomic, and metabolomic datasets were performed using the DIABLO framework implemented in the mixOmics R package (version 6.30.0) (27). DIABLO modelling was conducted on datasets that had undergone systematic, platform-specific quality control and missingness filtering prior to integrative analysis, as described above. Normalisation strategies were applied separately by data type as described above. DIABLO models were fitted with two latent components and a full design matrix linking all blocks, thereby maximising inter-omics covariance while discriminating between baseline and post-vaccination groups.

The classification performance of the prespecified models was assessed using participant-grouped cross-validation, ensuring that all longitudinal samples from the same participant remained within the same training or test partition. Repeated five-fold grouped cross-validation was used for the Pfizer-BioNTech and Moderna cohorts, whereas leave-one-participant-out validation was used for the smaller AstraZeneca cohort. Performance was evaluated using balanced error rate, balanced accuracy, overall accuracy, sensitivity, specificity, F1 score, confusion matrices, and out-of-fold AUROC. Potential overfitting was assessed by comparing full-cohort and cross-validated performance and through participant-wise label-permutation testing. Feature robustness was evaluated using selection frequency, loading-direction consistency, and Jaccard similarity across resampled models. A nested cross-validation sensitivity analysis was additionally performed to determine whether alternative numbers of components or retained variables materially improved model performance. Comprehensive DIABLO validation metrics can be found in the **Supplementary Methods**.

### Candidate biomarker identification and ROC analysis

Candidate biomarkers were defined as DIABLO-selected variables with non-zero loadings on the latent components. Inter-block structure and feature contributions were inspected using sample/variable plots, loadings, network diagrams, circos plots, and correlation heatmaps. Platform specificity was assessed by cross-platform comparison of expression profiles and visual inspection of time-course trajectories to identify patterns unique in direction and/or magnitude to a single vaccine, thereby distinguishing vaccine-specific responses from general vaccination effects. Discriminatory performance of each candidate was evaluated by receiver-operating-characteristic (ROC) analysis with pROC, using baseline versus 2wk-2nd as the classification task within each platform; areas under the ROC curve (AUROC) and 95% confidence intervals were computed via DeLong’s method (CIs omitted for AUROC = 1). ROC analyses were exploratory and performed within the discovery cohort.

## Results

### Normalisation benchmarking outcome

We benchmarked six preprocessing pipelines on a common, two-stage feature-filtered matrix for each omics layer and vaccine. A prespecified composite criterion, combining variance captured by PC1–PC3 with a centroid-separation metric calculated in a design-aware manner identified a top pipeline for each {vaccine, omics} block (**Table 3**). These block-specific choices were used to generate all matrices for downstream analyses, including PCAs. Robustness checks with the next-ranked pipeline per block produced qualitatively similar patterns and effect sizes (**Supplementary Figures S2-23**). We did not enforce a single normalisation across all datasets because distributional properties and censoring differ by omics layer and platform; the data-driven benchmarking avoided sub-optimal, one-size-fits-all processing. Following optimal preprocessing, we retained 244–255 proteins, 1339–1347 lipids, and 487 metabolites across all vaccine datasets (see **Supplementary Table S1** for full counts). To ensure that this lack of platform separation was not due to technical inconsistencies at baseline, we specifically compared baseline samples across platforms post-normalisation. These analyses confirmed that the baseline molecular landscape was consistent across vaccine groups (**Supplementary Figure S1**), supporting the validity of downstream comparisons. Baseline demographic characteristics were also compared across vaccine groups. Sex distribution was balanced, but a statistically significant age difference was observed, with the AstraZeneca group skewing older, reflecting age-based vaccination recommendations at the time of rollout (**Supplementary Figure S24**). These differences are considered when interpreting molecular responses.

**Table 3:** Selected normalisation strategies by vaccine and omics layer.

Vaccine	Proteomics	Lipidomics	Metabolomics
AstraZeneca	Default	Default	Complete
Moderna	Default	Quantile Z-score	Quantile Z-score
Pfizer	Complete	Complete	Quantile Z-score

### Unsupervised clustering reveals minimal grouping based on vaccine platform

Unsupervised PCA clustering of all vaccine platforms revealed minimal grouping (**Supplementary Method**). Platform-specific DE however, revealed that vaccines triggered extensive and highly directional molecular changes that were not apparent through unsupervised dimensionality reduction, with striking differences in both magnitude and directionality of response between platforms.

### Extensive molecular changes following Pfizer vaccination

Pfizer vaccination was associated with extensive molecular changes across all the three omics layers. Differential expression analysis comparing baseline to 2wk-2nd dose revealed widespread decreases in lipid abundance, bidirectional metabolite changes, and predominantly increased abundance of immunoglobulin- and complement-associated proteins.

#### Broad decreases across multiple lipid classes

Lipidomic profiling identified 1,347 lipid species, of which 872 (65%) were significantly altered (FDR < 0.05), including 852 downregulated and only 20 upregulated features (**Figure 1A**, **Supplementary Table S2**). Despite this broad suppression, only 206 species could be annotated, indicating that the lipid response is dominated by uncharacterised or poorly annotated compounds. Widespread lipid decreases spanned multiple structural and signalling lipid classes among the annotated compounds. Structural membrane-associated lipids showed coordinated reductions, including phosphatidylcholines (PC; 55 species, log_2_FC = −0.96), sphingomyelins (SM; 43 species, −0.98), lysophosphatidylcholines (LPC; 16 species, −0.95), and lysophosphatidylethanolamines (LPE; 3 species, −0.97). Ceramides (Cer; 17 species, −1.03), diacylglycerols (DG; 9 species, −1.02), and acylcarnitines (ACar; 12 species, −1.01) were also significantly decreased. Hexosylceramides (HexCer; 6 species, −1.31) and cardiolipins (CL; 17 species, −1.18) were among the most strongly decreased annotated lipid classes. Cholesteryl esters (CE; 4 species, −0.85) were similarly reduced. In contrast, fatty acids (FA; 2 species) showed increased abundance (log_2_FC = +0.73). Overall, Pfizer vaccination was associated with broad and coordinated changes in the abundance of multiple lipid classes; the functional processes underlying these changes cannot be established from plasma lipid abundance measurements alone.

**Figure 1:**
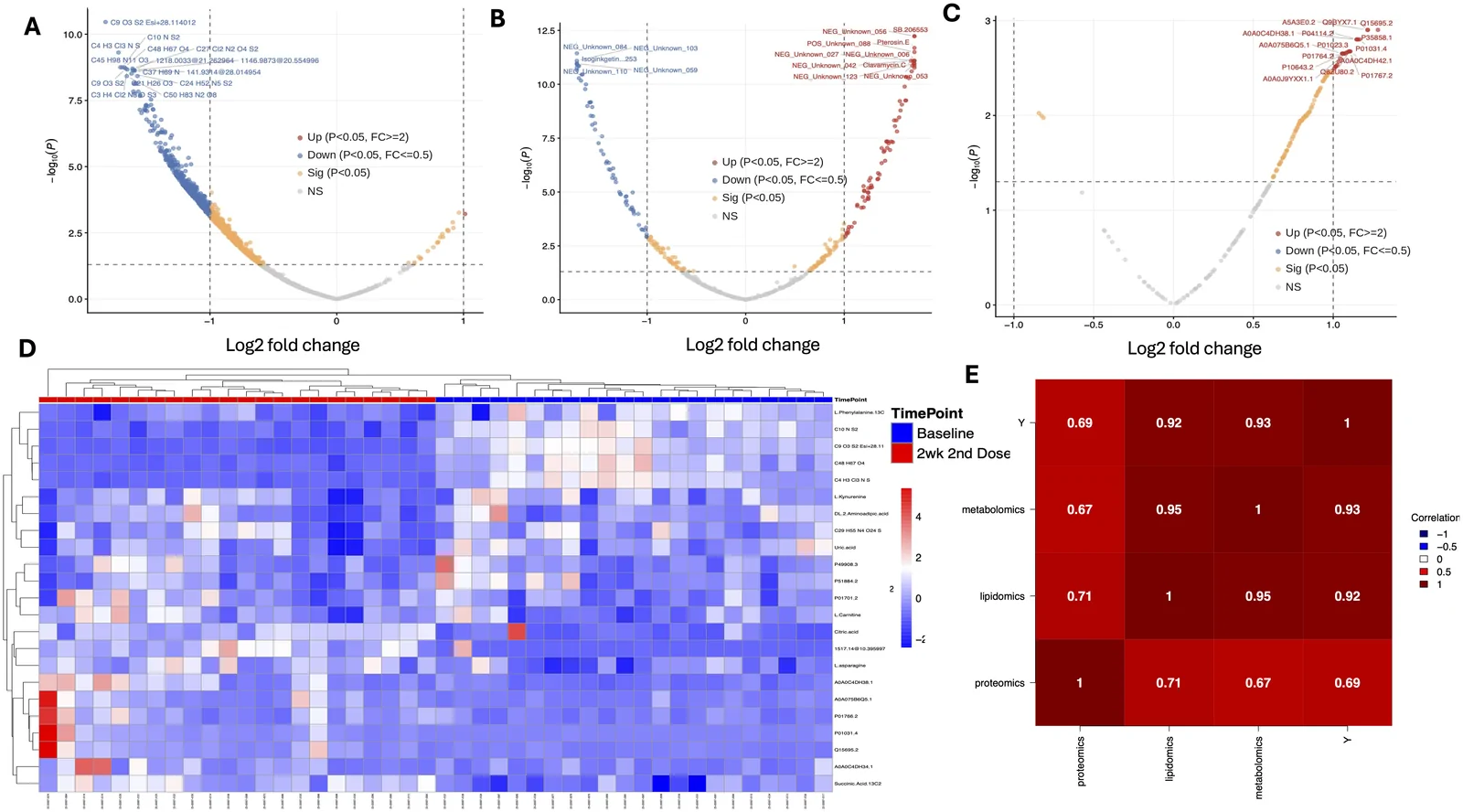
Integrated multi-omics analysis of the Pfizer-BioNTech vaccine response. **(A–C)** Volcano plots of differential abundance analysis showing log_2_ fold change (x-axis) versus –log_10_ adjusted p-value (y-axis) for significantly altered **(A)** lipids, **(B)** metabolites, and **(C)** proteins at 2 weeks post–dose 2 compared to baseline. Red points indicate features passing FDR <0.05 and |log_2_FC| >0.5. **(D)** Heatmap of selected discriminant features across longitudinal Pfizer timepoints, scaled to z-scores, showing temporal patterns of lipid, metabolite, and protein abundance following vaccination. **(E)** Multi-omics correlation heatmap of latent components showing strong inter-omics correlations across lipidomics, metabolomics, and proteomics blocks.

#### Bidirectional metabolomic abundance changes

Metabolomic profiling following Pfizer vaccination identified 241 significantly altered plasma metabolites (FDR < 0.05), comprising 112 downregulated and 129 upregulated features, indicating a broadly bidirectional metabolic response (**Figure 1B**, **Supplementary Table S3**). These changes involved metabolite classes, including amino-acid, organic-acid, carbohydrate, and fatty-acid-associated features. Notably, more than half of the significant metabolites were unannotated, including several of the most statistically significant features (e.g., NEG_Unknown_010; log_2_FC = –1.71, adj. p = 7.1 × 10^-11^), highlighting the contribution of currently uncharacterised small molecules to the vaccine-associated systemic response. Among annotated metabolites, amino acid-related features showed some of the most consistent alterations. Aromatic amino acids were markedly decreased, including L-phenylalanine (log_2_FC = –1.22), its isotope-labelled variant L-phenylalanine-^13^C (–1.32), and L-tryptophan (–1.21). Branched-chain amino acids (L-leucine and L-isoleucine; –0.65 each) and L-aspartic acid (–1.05) were similarly decreased. In contrast, L-asparagine (+0.98), L-serine (+0.67), and taurine (+0.79), were significantly increased. Trichotomine exhibited one of the strongest increases (+1.63), alongside several increased unannotated features (e.g., POS_Unknown_088; +1.71). Central carbon metabolism also showed differential abundance across several metabolites rather than a uniform directional response. Organic acids such as glyceric acid (–1.01) and L-hydroxyglutaric acid (–0.96) were decreased, while the TCA-cycle-associated metabolite succinic acid-^13^C_2_ was increased (+1.28). In parallel, carbohydrate-related metabolites and glycosylation intermediates, including D-maltose (+1.00), cellobiose (+0.79), and N-acetyl-α-D-glucosamine-1-phosphate (+0.66), were increased. Long-chain and very-long-chain saturated fatty acids in the metabolomics dataset, including palmitic acid (–0.86), cerotic acid (–0.96), melissic acid (–0.93), and octacosanoic acid (–1.00), showed decreased abundance. In contrast, the lipidomics dataset revealed increased abundance of a small subset of free fatty acids (mean log_2_FC = +0.73), demonstrating differing abundance patterns between fatty-acid-associated features measured across the metabolomic and lipidomic datasets. Finally, several xenobiotic and food-associated metabolites exhibited large-magnitude changes, including drug glucuronides, flavonoids (e.g., isoginkgetin), and capsinoids (e.g., dihydrocapsiate). The biological basis of these changes cannot be determined from plasma metabolite abundance alone and will require further functional investigation.

#### Proteomic signature dominated by immunoglobulin and complement-associated proteins

Proteomic analysis identified 144 significantly altered proteins, with the overwhelming majority upregulated (141 increased vs 3 decreased; **Figure 1C**, **Supplementary Table S4**). The dominant features included immunoglobulin variable and constant regions, consistent with a strong immunoglobulin-associated plasma protein response following vaccination. Increased heavy chain variants included IGHV3-66 (+1.11), IGHV5-51 (+1.15), and IGHV3-49 (+1.03), along with light chains IGKV3D-15 (+1.02) and IGLV3-16 (+0.83). Constant regions of IgM and IgG subclasses were similarly increased. Multiple complement-associated proteins also showed increased abundance, including classical and terminal pathway components C5 (+1.16), C3 (+1.04), C2 (+1.06), C7 (+1.08), and C9 (+0.95), together with regulatory proteins C4BPA (+1.01), CFH (+0.92), and CFI (+0.67). Other immune-associated proteins such as A2M (+1.10), SERPIND1 (+1.07), and APOB (+1.15) were also increased. Together, these findings demonstrate a coordinated increase in the abundance of immunoglobulin and complement-associated plasma proteins following Pfizer vaccination.

#### Multivariate integration identifies a cross-omic molecular signature

DIABLO-based integration of lipidomic, metabolomic, and proteomic data revealed a multivariate molecular signature distinguishing baseline and post-vaccination states (**Figures 1D, E**, **Supplementary Figure 27**). Lipidomics contributed strongly to the model, with most discriminant features showing negative loadings associated with the baseline condition, consistent with the observed decreases in lipid abundance following vaccination (**Supplementary Figure 27A**). A smaller subset of lipids showed positive loadings at the post-vaccination timepoint, reflecting selective upregulation of specific lipid species. Metabolomic features showed a balanced loading pattern, with three discriminant features positively associated with the post-vaccination timepoint and three negatively associated (**Supplementary Figure 27B**). This distribution mirrors the directionality observed in the univariate DE analysis (112 downregulated, 129 upregulated), reinforcing the consistency of the metabolic signature across multivariate and univariate approaches. The DIABLO proteomic component revealed a coherent molecular signature associated with the post-vaccination state (**Supplementary Figure 27C**). All proteins selected as discriminant features displayed positive loadings at the 2-week timepoint, showing positive loading across this layer. These features included key plasma proteins involved in lipid transport and immune regulation, such as apolipoprotein A-I (APOA1), apolipoprotein A-IV (APOA4), apolipoprotein F (APOF), serum amyloid P-component (APCS), zinc-alpha-2-glycoprotein (AZGP1), complement factor H-related protein 5 (CFHR5), and extracellular matrix protein 1 (ECM1). Importantly, these proteins were also among those significantly upregulated in the univariate DE analysis, reflecting strong consistency across statistical approaches. The concordant direction of these proteomic features contributed to the separation of baseline and post-vaccination samples in the integrated model without implying functional activation of the associated biological pathways. Cross-platform correlations were high, with lipidomics and metabolomics showing the strongest association (r = 0.95), followed by proteomics with lipidomics (r = 0.71) and metabolomics (r = 0.67) (**Figure 1E**, **Supplementary Figure 26D**). These correlations indicate strong covariance among the molecular profiles across omics layers. The integrative heatmap (**Figure 1D**) demonstrated clear sample stratification by timepoint, driven by consistent upregulation of immune-related proteins (e.g., C5, APOB, immunoglobulin heavy chains) and suppression of lipids and metabolites. Together, these results identify a coordinated multi-omic signature associated with Pfizer vaccination, characterised by widespread lipid decreases, bidirectional metabolite changes, and increased abundance of several immune-associated plasma proteins.

### Moderna vaccination shows lipidomic and metabolomic changes

Moderna vaccination was associated with extensive lipidomic and metabolomic changes. While univariate proteomic analysis did not identify significant changes, multivariate integration identified a molecular signature spanning all three omics layers.

#### Predominantly increased abundance across several lipid classes

Lipidomic profiling identified 354 significantly altered lipid species 2wk-2nd Moderna dose (26.3% of 1,346 profiled; FDR < 0.05), with a predominant increase in abundance (307 upregulated, 47 downregulated) (**Figure 2A**, **Supplementary Table S5**). Only 48 of these lipids were successfully annotated, highlighting the contribution of structurally unresolved compounds to the vaccine response. Among known lipid classes, acylcarnitines (ACar) were notably elevated (6 species; mean log_2_FC = +1.01). Lysophosphatidylcholines (LPCs) were also increased (7 species; +0.84). Conversely, triacylglycerols (TAGs) showed consistent depletion (8 species; –0.61), while Ceramides (Cer) also showed an overall decrease (7 species; –0.57). Cardiolipins (CLs) showed increased abundance (7 species; mean log_2_FC = +0.93). Overall, Moderna vaccination was associated with a predominantly increased lipidomic profile across several annotated lipid classes, contrasting with the broad lipid decreases observed following Pfizer vaccination. These abundance changes do not directly establish alterations in mitochondrial activity, β-oxidation, membrane remodelling, apoptotic signalling, or other functional metabolic processes.

**Figure 2:**
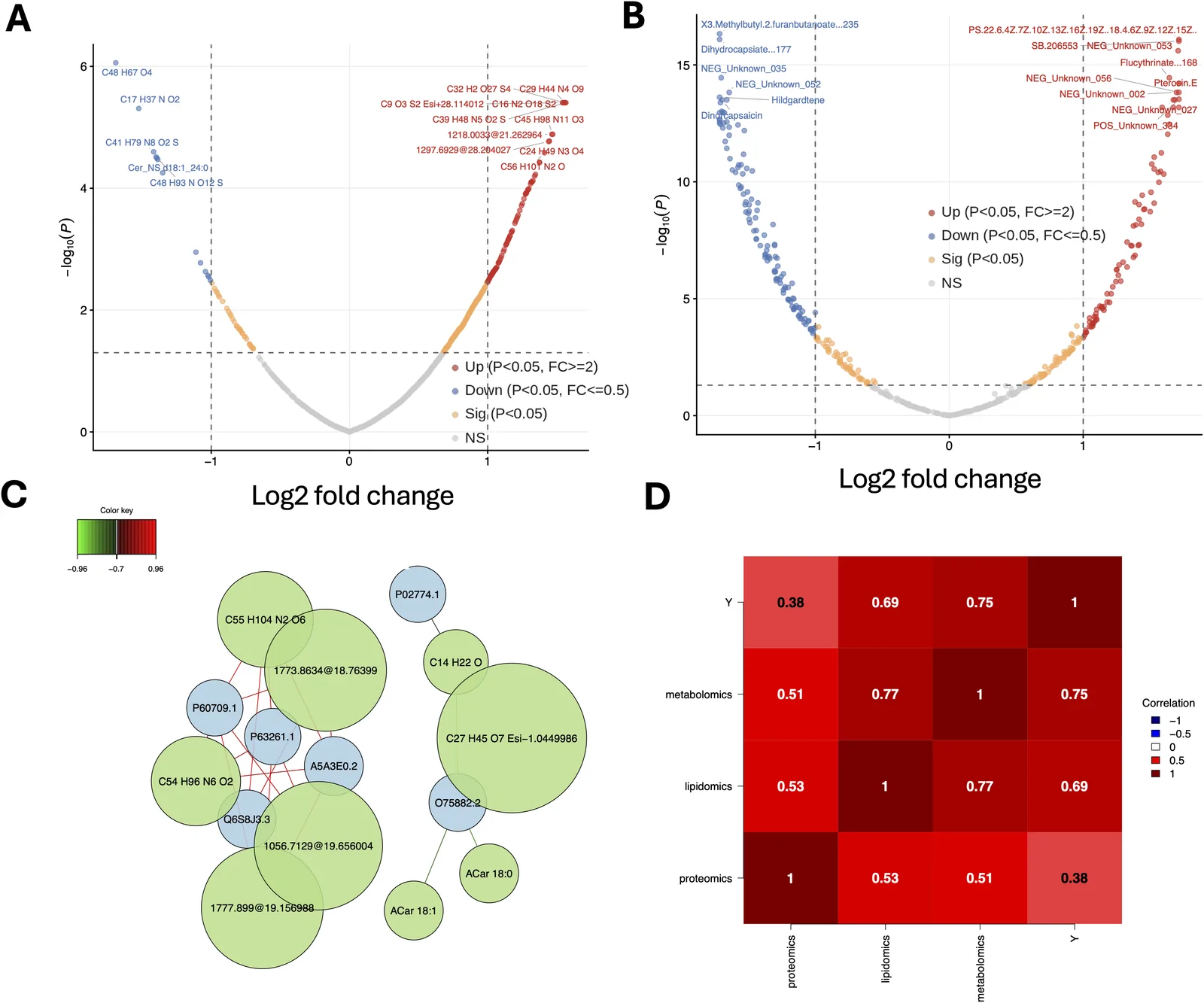
Moderna integrated multi-omics analysis of vaccine-induced responses. **(A, B)** Volcano plots of differential abundance analysis showing log_2_ fold change (x-axis) versus –log_10_ adjusted p-value (y-axis) for significantly altered features in **(A)** lipids and **(B)** metabolites. Red points indicate features passing FDR <0.05 and |log_2_FC| >0.5. **(C)** DIABLO network plot showing cross-omic associations between selected features from lipidomics (green nodes) and proteomics (blue nodes). Node size reflects feature importance (loading weight) in Component 1, while edge color indicates correlation direction (red: positive; green: negative; |r| ≥ 0.7). The network highlights a central lipid–protein module associated with the post-vaccination state. **(D)** Multi-omics correlation heatmap of latent components showing pairwise correlation coefficients across lipidomics, metabolomics, and proteomics blocks.

#### Bidirectional metabolomic abundance changes involving amino acids, organic acids, and xenobiotics

Metabolomic profiling revealed 315 significantly altered metabolites out of 487 measured (FDR < 0.05), of which 98 were annotated (**Figure 2B**, **Supplementary Table S6**). Unlike the lipidome, the metabolome showed bidirectional changes, with increases and decreases across distinct molecular classes. Multiple amino acids and derivatives were increased, including L-leucine, L-isoleucine, L-phenylalanine, L-methionine, L-tyrosine, L-glutamine, L-citrulline, and L-carnitine, with mean log_2_FC values between +0.7 and +1.1. Several small peptides were also increased. In contrast, select amino acid-related intermediates (e.g., hypoxanthine, acronycidine, moschamine) were decreased. Increases were also observed in organic acids (e.g., lactic acid, hydroxyglutaric acid) and carbohydrate intermediates (e.g., fructose-6-phosphate, N-acetyl-glucosamine-6-phosphate). Very-long-chain fatty acids such as cerotic acid and melissic acid were decreased, demonstrating additional changes in fatty-acid-associated metabolites alongside those observed in the lipidomics dataset. Several xenobiotic and environmental metabolites showed large-magnitude changes (|log_2_FC| > 1.5), including drug conjugates (e.g., fluconazole- and rosuvastatin-glucuronides) and plant-derived molecules (e.g., quercetin derivatives, isoginkgetin). Many of the most altered features remained unannotated, again pointing to a substantial contribution of unresolved small molecules to the systemic vaccine response.

#### Multivariate integration identifies a cross-omic molecular signature

Although no proteins were significant in univariate proteomic analysis, multivariate integration using DIABLO revealed a marked molecular signature distinguishing pre and post-vaccination states (**Supplementary Figures S28A-C**). The lipidomic block contributed strongly to the separation, driven by long-chain acylcarnitines (ACar 18:0, ACar 18:1) and complex lipids (e.g., C27H45O7, C14H22O), most of which were decreased in the post-vaccination state (**Supplementary Figure S28A**). The metabolomic block showed discriminant loadings for amino acids and nucleotide metabolites (e.g., L-isoleucine, L-leucine, guanosine), which despite being increased in DE analysis were negatively associated with the post-vaccine condition in the multivariate model (**Supplementary Figure S28B**). The basis of this divergence cannot be determined from the present analyses. The proteomic component, although not individually significant in DE testing, contributed to the DIABLO signature through features such as fibrinogen gamma chain (P02679), 14-3–3 protein epsilon (P02774), oxidoreductase TXNDC17 (O75882.2), and immunoglobulin fragments (e.g., P01859, A0A0C4DH34.1, **Supplementary Figure S28C**). These proteins showed strong correlations with lipid and metabolite features, demonstrating cross-omic associations among selected features. This highlights the utility of multivariate models in identifying proteomic features contributing to multivariate discrimination. DIABLO network analysis revealed associations between lipidomic and proteomic features, with a central cluster of complex lipids (e.g., C55H104N2O6, C54H96N6O2, 1773.8634@18.76) positively correlated with immune-related proteins including actin (P60709.1), 14-3–3 epsilon (P63261.1), and immunoglobulin fragments (A5A3E0.2) (**Supplementary Figure 28C**). In contrast, TXNDC17, a redox-regulating protein, was negatively correlated with acylcarnitines (ACar 18:0, ACar 18:1). These correlations describe statistical associations between molecular features and do not establish direct regulatory or functional relationships. Global correlation analysis showed inter-omic associations: lipidomics and metabolomics showed the strongest inter-omic association (r = 0.77), with moderate correlations between each and proteomics (r = 0.51 and 0.53, respectively) (**Figure 2D**). A Circos plot of high-confidence cross-omic connections (|r| ≥ 0.7) revealed a predominance of positive correlations (**Supplementary Figure S28D**).

Together, these analyses identify a correlated multi-omic signature associated with Moderna vaccination; the functional relationships among these molecular features require further investigation.

### AstraZeneca vaccination is associated with subtle molecular changes

AstraZeneca vaccination was associated with moderate molecular changes, primarily observed in the metabolome, with weaker signals detected in the lipidome and proteome. Differential expression and multivariate integration analyses revealed cross-omic associations, despite the limited statistical power of the small sample size (n = 7).

#### Modest bidirectional changes in plasma metabolites

Differential expression analysis identified 234 significantly altered metabolites out of 487 measured (FDR < 0.05), comprising 139 upregulated and 95 downregulated features (**Figure 3A**, **Supplementary Table S7**). The magnitude of change ranged from log_2_FC = –1.92 to +1.92, with 47.9% (112 features) remaining unannotated, underscoring the contribution of unknown molecules to the vaccine response. Top upregulated features included SB-206553 (+1.92), POS_Unknown_334 (+1.91), and Pterosin E (+1.90), while decreased features included Didodecyl thiobispropanoate (–1.92), β-santalic acid (–1.91), and 3-methylbutyl 2-furanbutanoate (–1.90). Among annotated compounds, several fatty acid associated metabolites changed in abundance, with decrease of palmitic acid (–1.81) and eicosanedioic acid (–1.67), and increase of tridecanoic acid (+1.29). Multiple phospholipids were significantly altered, including PE (11:0/9:0), PA (20:5/22:6), PI (17:1/17:1), and PS (22:6/18:4). In the peptide pool, dipeptides and tripeptides such as Glu-Trp-Glu, Trp-Trp-Lys, and Pro-Pro-Lys were differentially expressed, while Tyr-Ile-OH variants showed mixed directional responses. Together, these findings indicate a heterogeneous metabolomic response following AstraZeneca vaccination without establishing the functional processes underlying these abundance changes.

**Figure 3:**
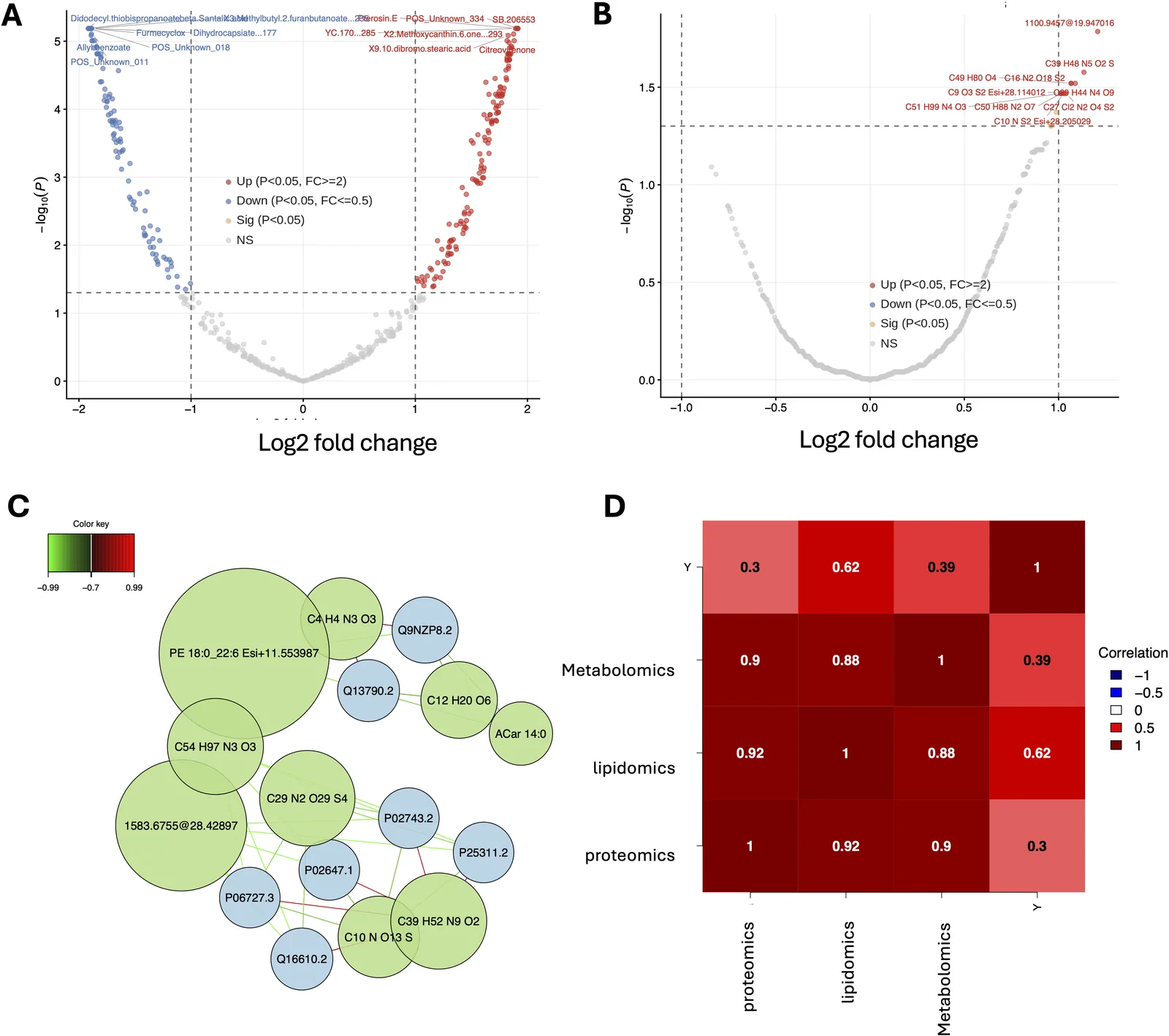
DIABLO-based multi-omics integration of the AstraZeneca vaccine response. **(A)** Volcano plots of differential abundance analysis showing log_2_ fold change (x-axis) versus –log_10_ adjusted p-value (y-axis) for significantly altered features in metabolites. Red points indicate features passing FDR <0.05 and |log_2_FC| >0.5 **(B)** Multi-omics correlation heatmap of latent components showing pairwise correlation coefficients across lipidomics, metabolomics, and proteomics blocks. **(C)** DIABLO network plot showing cross-omic associations between selected features from lipidomics (green nodes) and proteomics (blue nodes). Node size reflects feature importance (loading weight) in Component 1, while edge color indicates correlation direction (red: positive; green: negative; |r| ≥ 0.7). The network highlights a central lipid–protein module associated with the post-vaccination state. **(D)** Multi-omics correlation heatmap of latent components showing pairwise correlation coefficients across lipidomics, metabolomics, and proteomics blocks.

#### Limited univariate signals in lipidomics and proteomics

No protein features reached significance in the univariate analysis. In contrast, 14 lipid features met the prespecified significance criteria, all of which showed higher abundance at two weeks after dose 2 compared with baseline (**Figure 3B**, **Supplementary Table S8**). Most significant lipid features were structurally unresolved: 11 were unclassified, two were unmatched or assigned to other lipid classes, and one was annotated as a cardiolipin. Given the small AstraZeneca cohort (n = 7), these lipidomic findings should be considered exploratory.

#### Multivariate integration identifies a cross-omic signature

DIABLO-based integration resolved a multi-omic signature separating pre and post-vaccination samples (**Supplementary Figure 29**). Despite limited DE hits in lipidomics and proteomics, the correlation structure between omics blocks was strong: proteomics–lipidomics (r = 0.92), proteomics–metabolomics (r = 0.90), and lipidomics–metabolomics (r = 0.88) (**Figure 3D**). These correlations indicate strong covariance across omics layers. Discriminant features in the proteomic block included APOA1 (P02647), APOA4 (P06727), APOF (Q13790), APCS (P02743), AZGP1 (P25311), ECM1 (Q16610), and CFHR5 (Q9NZP8), all with positive loadings at the post-vaccination timepoint (**Figure 3C**). The lipidomic block contributed discriminant features such as C54_H97_N3_O3, C29_N2_O29_S4, and C39_H52_N9_O2, most showing negative loadings (**Supplementary Figure 29B**). In the metabolomics block, features such as N-acetyl-D-glucosamine-6-phosphate, succinic acid-^13^C_2_, N-acetyl-α-D-glucosamine-1-phosphate, L-asparagine, and creatine were positively loaded and contributed to sample separation (**Supplementary Figure 29C**). Network analysis of discriminant features (r ≥ 0.7) revealed modular cross-omic relationships (**Supplementary Figure 29D**). A central cluster linked apolipoproteins (APOA1, APOA4, APOF), key mediators of lipid transport and cholesterol metabolism, to lipid species including C29_N2_O29_S4, C10_N_O13_S, and C39_H52_N9_O2. APCS, a component of the pentraxin family involved in acute-phase response, and AZGP1, a lipid-mobilising factor linked to metabolic regulation, showed distinct associations with specific lipid features. ECM1 and CFHR5 were connected to additional molecular nodes within the network (**Supplementary Figure 29C**). Although AstraZeneca induced fewer univariate changes than mRNA vaccines, especially in proteomics and lipidomics, the DIABLO model captured a cross-omic molecular signature. Hierarchical clustering of discriminant features (**Supplementary Figure 29E**) showed clear separation between baseline and post-vaccination samples, particularly for metabolites such as succinic acid-^13^C_2_, L-asparagine, and N-acetylglucosamine derivatives. Given the small sample size (n = 7), these findings are preliminary and require validation in larger cohorts. Nonetheless, the strong inter-omic correlations and consistent sample stratification support the presence of a correlated cross-omic molecular signature associated with AstraZeneca vaccination.

Participant-grouped cross-validation supported discrimination between baseline and post-vaccination states, with BERs of 0.008, 0.039, 0.002, and 0.033 for Pfizer 2wk-2nd, Pfizer 6mo-2nd, Pfizer 2wk-3rd, and Moderna, respectively, with corresponding out-of-fold AUROCs of 1.000, 0.978, 0.994, and 0.992. AstraZeneca also showed strong discrimination (BER = 0; AUROC = 1.00), although this result should be interpreted cautiously given the small cohort size (n = 7). Permutation testing supported performance above chance, while feature-set stability was lower than classification stability, indicating that individual discriminant features should be interpreted cautiously (**Supplementary Figure S26**).

Sensitivity analyses using alternative preprocessing pipelines preserved the principal directional patterns across vaccine and omics comparisons, with particularly high concordance for Pfizer lipidomic and metabolomic responses and for Moderna and AstraZeneca metabolomics; greater variability was observed in exact feature-level significance calls for Moderna and AstraZeneca lipidomics and later Pfizer proteomic responses (**Supplementary Figure S26**).

### The Pfizer vaccine induces temporal multi-omic changes

Longitudinal multi-omic profiling of Pfizer vaccine recipients uncovered temporally distinct but coordinated molecular response patterns. By analysing lipids, polar metabolites, and proteins across three timepoints, we observed time-dependent abundance changes across all omics layers, with each compartment showing distinct temporal patterns (**Figure 4**).

**Figure 4:**
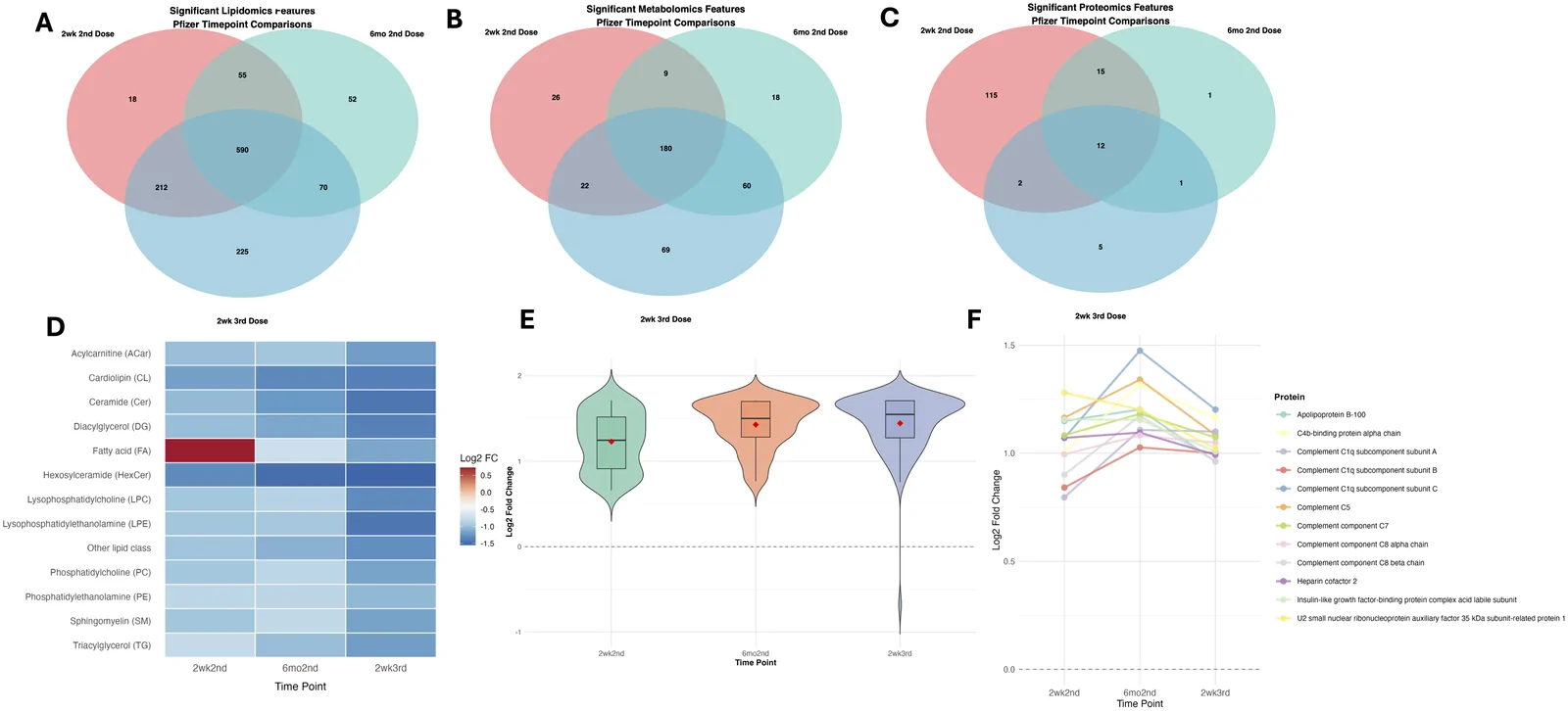
Overlap and temporal dynamics of vaccine-induced molecular features. **(A-C)** Venn diagrams showing overlap of significantly altered **(A)** lipids, **(B)** metabolites and **(C)** proteins, and across vaccine platforms. **(D)** Heatmap of lipid class showing normalised abundance patterns across timepoints. The colour scale reflects direction and magnitude of change, with red indicating increased abundance and blue indicating decreased abundance relative to baseline. **(E)** Violin plots show log2 fold change distributions for metabolites present at all three vaccination time points. Violin width indicates data density. Box plots (within violins) show interquartile range with median (black line). Red diamonds (♦) indicate mean values. Dashed line at y=0 represents no change. **(F)** Line plots of log_2_ fold change trajectories for most significant molecular features across timepoints (Baseline, 2wk 2^nd^, 6mo 2^nd^, and 2wk 3^rd^).

#### Progressive decreases in lipid abundance across Pfizer vaccination timepoints

Lipidomic profiling revealed extensive decrease in lipid abundance following Pfizer vaccination that remained prominent across all three sampled timepoints. At 2wk-2nd dose 872 lipid species were significantly altered comprising 852 with decreased and 20 with increased abundance (**Figure 4A**). At 6mo-2nd, 840 lipids were significantly altered comprising 825 with decreased and 15 with increased abundance, while at 2wk-3rd following booster vaccination 1,099 species were significantly altered, comprising 1,088 with decreased and 11 with increased abundance. Decreased abundance was observed across all major lipid classes, including phosphatidylcholines (40–66 species), sphingomyelins (24-59), and phosphatidylethanolamines (6–11) (**Figure 4D**). Ceramides showed progressively lower abundance from 2wk-2nd to 2wk-3rd (mean log_2_FC –1.03 to –1.44). Cardiolipins were consistently decreased (mean log_2_FC –1.18 to –1.38). Lysophospholipids, including LPCs (16–23 species) and LPEs (3 species), also showed decrease abundance. Free fatty acids showed a biphasic response: modest upregulation at 2wk-2nd (mean log_2_FC +0.73, 2 species), followed by decrease at later timepoints (mean log_2_FC –0.74 at 6mo-2nd; –1.15 at 2wk-3^rd^). Overall, these findings demonstrate persistent, predominantly decreased lipid abundance across the sampled Pfizer vaccination timepoints. (**Supplementary Tables S2, S9, S10**).

#### Pfizer vaccination shows sustained changes in polar metabolite abundance

Metabolomic profiling showed sustained changes in polar metabolite abundance following Pfizer vaccination. At 2wk-2nd, 241 metabolites were significantly altered (**Figure 4B**). At 6mo-2nd, 274 metabolites were significantly altered, increasing to 334 at 2wk-3rd. Violin plots (**Figure 4E**) showed a rightward shift in the distributions of metabolite effect estimates at the later sampled timepoints relative to 2wk-2nd, indicating a greater predominance of increased metabolite abundance. Responses were class-specific. Amino acids and derivatives showed mixed changes: branched-chain (L-isoleucine, L-leucine) and aromatic amino acids (L-phenylalanine, L-tryptophan) were consistently decreased, while several amino acid derivatives and small peptides showed increased abundance. Organic acids associated metabolites also showed heterogeneous changes, with decreases in selected dicarboxylic and fatty acids alongside increased abundance of hydroxy- and keto-acids. Sugar intermediates such as D-glucose-6-phosphate and D-sedoheptulose-7-phosphate were decreased at 2wk-3rd. Overall, polar metabolite changes showed a net predominance of increased abundance at the later time points. Notably, 183 metabolites were consistently altered across all three timepoints and 245 features were shared between 6mo-2nd and 2wk-3rd defining a persistent metabolic signature across the sampled time points (**Supplementary Tables S3, S11, S12**). These findings highlight divergent abundance patterns between lipid and polar metabolite compartments, with Pfizer vaccination showing persistent lipid decrease alongside predominantly increased polar metabolite abundance at later sampled timepoints.

#### Proteomic abundance patterns vary across Pfizer vaccination timepoints

Proteomic response to Pfizer vaccination showed distinct temporal patterns, with broad changes at 2wk-2nd, fewer differentially abundant proteins at 6mo-2nd, and renewed changes in selected proteins at 2wk-3rd. While the early phase featured extensive increase in abundance, particularly among immunoglobulin and complement components, as described earlier, longitudinal analysis revealed a clear shift in proteomic profiles over time. These changes are visualised in a line plot of log_2_FC trajectories for individual proteins across timepoints (**Figure 4F**), which illustrates time-dependent changes in protein abundance and the sustained elevation of select components such as complement proteins. By 6mo-2nd, the number of differentially abundant proteins had contracted substantially, with persistent elevation of regulatory complement components (e.g., C1q subunit C, C4b-binding protein alpha chain). At 2wk-3rd, selected terminal complement-associated proteins (e.g., C5, C7, C8) again showed increased abundance, whereas the broad increase in immunoglobulin-associated proteins observed after the second dose was not reproduced. This progression is supported by a Venn diagram showing the overlap of significant proteomic features across timepoints (**Figure 4C**), which highlights a core set of 12 shared proteins, largely involved in complement activation, and distinct sets unique to each phase of the response (**Supplementary Tables S4, S13, S14**).

Together, these findings show temporally distinct multi-omic abundance patterns following Pfizer vaccination, characterised by persistent lipid decreases, sustained changes in polar metabolite abundance, and time-dependent changes in immunoglobulin and complement-associated proteins.

### Pfizer and Moderna vaccines induce opposite multi-omic responses

To understand common and divergent features of mRNA vaccine responses, we directly compared molecular signatures associated with Moderna and Pfizer. Despite shared use of mRNA technology and lipid nanoparticle platforms, the two vaccines showed distinct metabolic and lipidomic response patterns. While metabolomic profiles revealed a largely overlapping core response, lipidomic changes showed marked divergence, with opposing directional shifts across nearly all major lipid classes (**Figure 5**).

**Figure 5:**
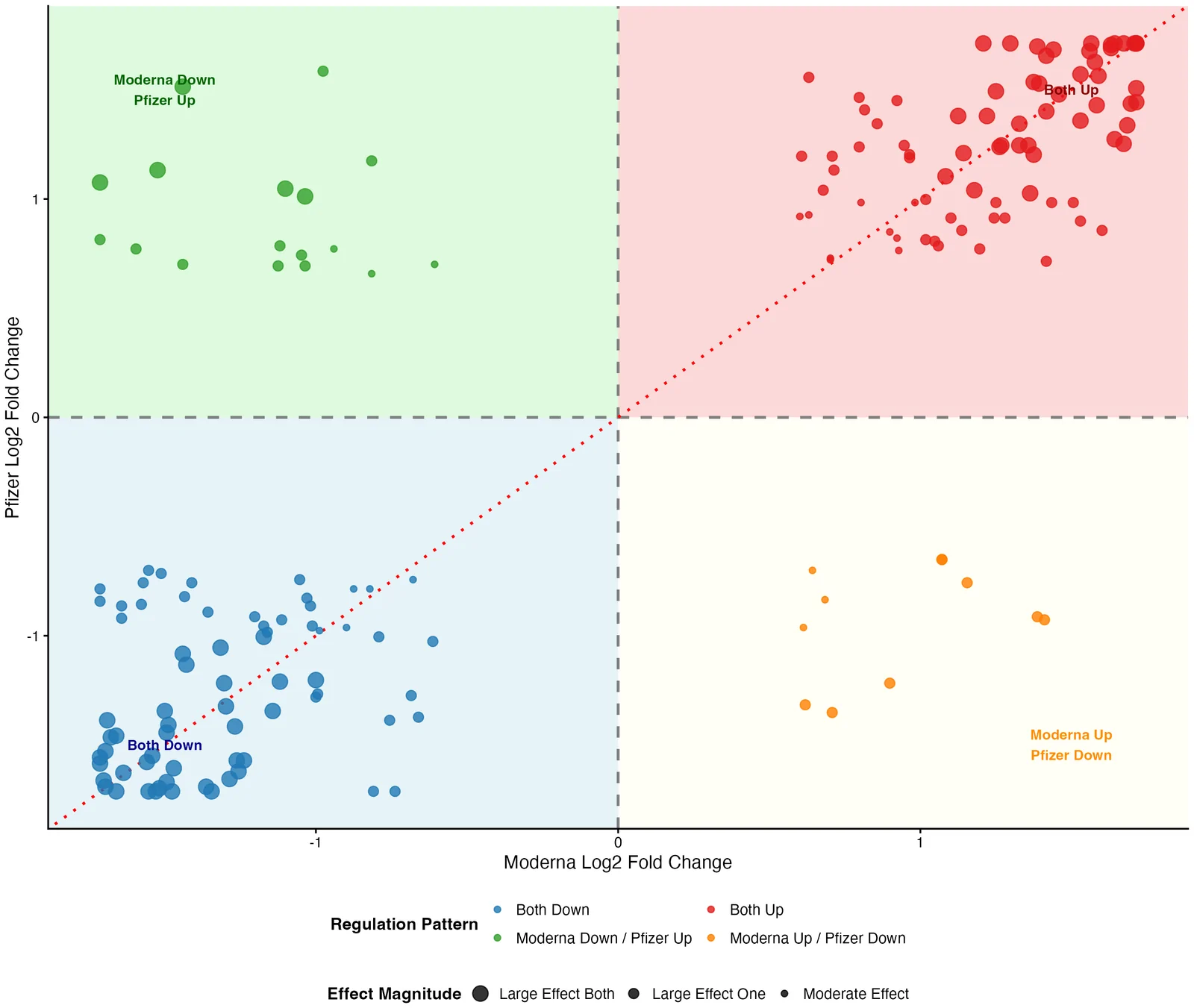
Comparison of metabolite responses to Moderna vs Pfizer vaccination. Scatter plot shows log_2_ fold changes in shared metabolites between Moderna (x-axis) and Pfizer (y-axis) at 2 weeks post-second dose. Colours indicate regulation pattern: red (both up), blue (both down), green (Pfizer up/Moderna down), orange (Moderna up/Pfizer down). Dot size reflects effect magnitude. Dashed lines mark no change; the diagonal indicates equal regulation in both vaccines.

#### Conserved core metabolomic responses to Moderna and Pfizer vaccination

We compared plasma metabolites significantly altered according to the predefined significance criteria by both Moderna and Pfizer at 2 weeks post-second dose. A total of 181 metabolites were significantly altered in both vaccine cohorts. Among these, 84% (n = 152) exhibited concordant regulation, including 81 metabolites upregulated and 71 downregulated across both groups (**Figure 5**). These changes spanned diverse compound classes, with several showing large effect sizes (|log_2_FC| > 1.5) in both cohorts. Notable examples included Pterosin E and Citreovirenone (both increased), and Dihydrocapsiate, Isoginkgetin, and Dinorcapsaicin (consistently decreased), alongside numerous unannotated high-magnitude features (e.g., NEG_Unknown_053). A subset of 29 metabolites (16%) showed discordant directionality between the two cohorts. For example, L-phenylalanine was upregulated by Moderna (log_2_FC = +0.90) but downregulated by Pfizer (–1.22). Conversely, Hildgardtene showed strong downregulation with Moderna (–1.71) but upregulation with Pfizer (+1.08). Several of these discordant features also showed large effect sizes. Overall, these identify a shared pattern of metabolite abundance changes across the two mRNA vaccine cohorts, together with a smaller subset of discordant features.

#### Opposing lipidomic abundance patterns following Pfizer and Moderna vaccination

Lipidomic profiling revealed markedly different lipid abundance patterns between Pfizer and Moderna vaccines (**Figure 6**). Pfizer vaccination was associated with widespread decrease in lipid abundance with 872 significantly altered lipid species, of which 852 compounds, 97.7%) showed decreased abundance compared with baseline. Decreases were observed across multiple annotated lipid classes, including sphingomyelins, phosphatidylcholines, phosphatidylethanolamines, lysophospholipids, triacylglycerols, and diacylglycerols. By contrast, Moderna vaccination showed predominantly increased lipid abundance, with 354 significantly altered lipid species, of which 307 (86.7%) were increased and 47 were decreased, with a mean model-derived effect log_2_FC of +0.67 across significant features. Among annotated classes showing increased mean abundance were Acylcarnitine (6 species; mean log_2_FC = +1.09), sphingomyelins (2 species; mean log_2_FC = +0.86), and phosphatidylcholines (2 species; log_2_FC = +0.77). Direct comparison of the 249 lipid species that were significantly altered in both vaccine groups revealed that 206 (82.7%) showed opposite directionality: systematically decreased in Pfizer recipients but increased in abundance in Moderna recipients. This opposition was evident across all major lipid classes. Cardiolipin showed particularly striking divergence, with 7 species decreased by Pfizer (mean log_2_FC = -1.1) while 7 species were increased by Moderna (mean log_2_FC = +0.93). Similarly, phosphatidylcholines, and acylcarnitine consistently displayed inverse patterns between platforms. These opposing lipidomic profiles indicate that, despite their shared mRNA technology and lipid nanoparticle delivery platforms, Pfizer and Moderna vaccination are associated with distinct patterns of lipid abundance. Pfizer showed predominantly decreased lipid levels, whereas Moderna showed a predominantly increased lipidomic profile. These differences may reflect platform-specific effects on lipid metabolism or membrane-associated processes, but the underlying functional mechanisms cannot be established from plasma lipid abundance measurements alone.

**Figure 6:**
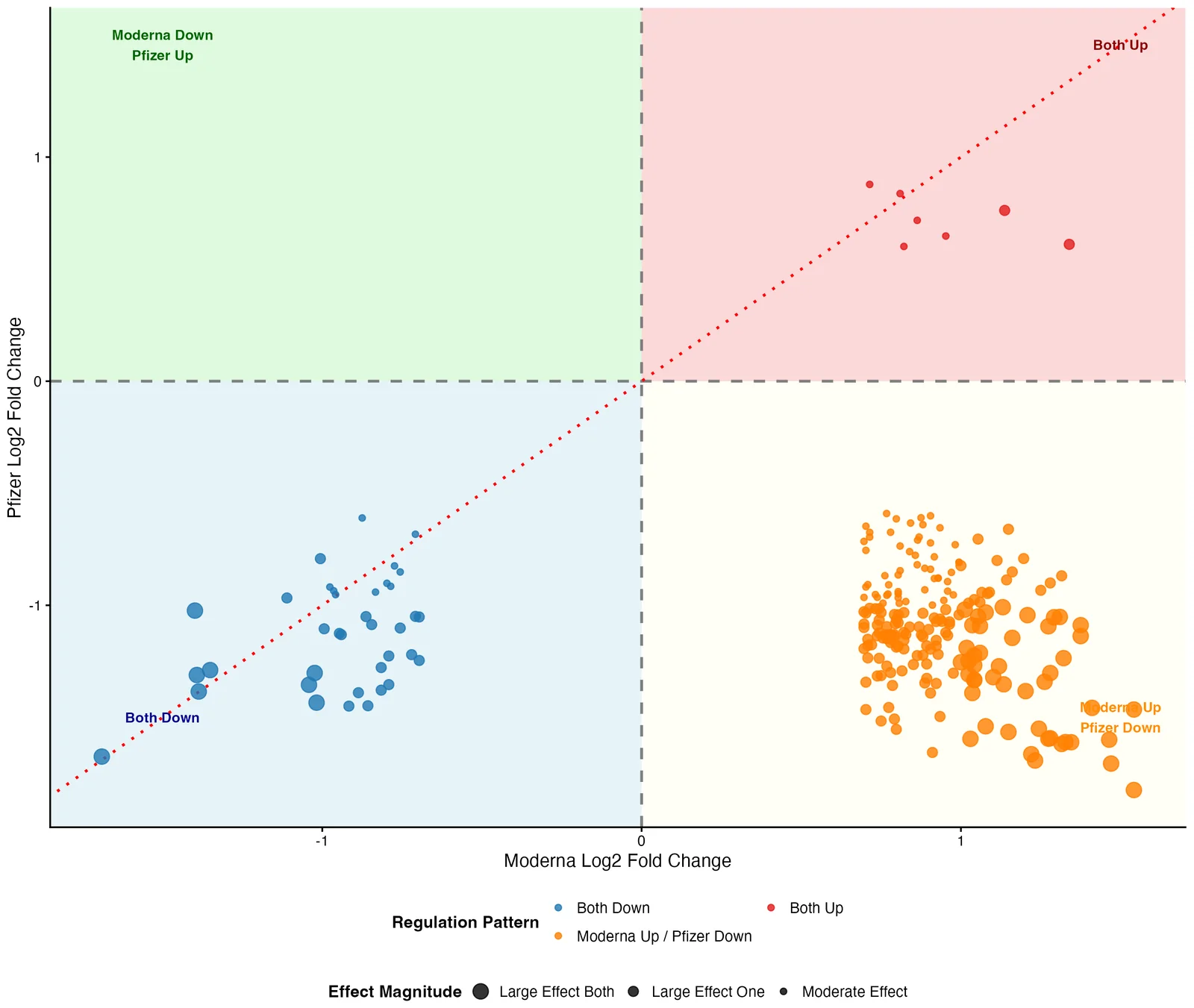
Comparison of lipid responses to Moderna vs Pfizer vaccination. Scatter plot shows log_2_ fold changes in shared lipids between Moderna (x-axis) and Pfizer (y-axis) at 2 weeks post-second dose. Colors indicate regulation pattern: red (both up), blue (both down), green (Pfizer up/Moderna down), orange (Moderna up/Pfizer down). Dot size reflects effect magnitude. Dashed lines mark no change; the diagonal indicates equal regulation in both vaccines.

Despite their shared mRNA platform, Pfizer and Moderna vaccines showed markedly divergent lipidomic responses but largely concordant metabolite abundance patterns. Moderna was associated with predominantly increased lipid abundance, whereas Pfizer showed widespread lipid decrease. In contrast, 84% of significantly altered metabolites showed concordant directionality, defining a common set of metabolite abundance changes across the two cohorts. These observations identify distinct lipidomic profiles within a partially shared metabolomic response; the biological mechanisms underlying these differences require further investigation.

### Platform-specific biomarker candidates

DIABLO feature selection identified molecular features that were evaluated as candidate platform-associated biomarkers within the present discovery cohort. Their potential utility requires validation in independent cohorts.

### Astrazeneca and Moderna candidate biomarker

Remarkably, no candidate biomarkers meeting the stringent selection criteria were identified for Astrazeneca and Moderna vaccination across any omics layer. This finding suggests that Astrazeneca’s and Moderna’s molecular signature, while robust in magnitude, may be composed of responses that are either shared with other vaccines or represent amplified versions of common vaccination responses rather than distinct molecular signatures.

### Pfizer candidate biomarkers

Pfizer vaccination was characterised by the most comprehensive candidate biomarker signature, spanning all three omics layers and demonstrating temporal persistence. Four lipid species emerged as potential candidate biomarkers with vaccine-specific expression patterns that remained detectable across multiple timepoints. Lipid candidate biomarkers included 1517.14@10.395997 (**Figure 7A**), which showed a marked increase uniquely in Pfizer recipients while remaining low in other vaccine groups, and achieved high within-cohort discriminatory performance (AUROC = 0.96, 95% CI: 0.87–1.00). C_10_NS_2_ (**Figure 7B**), identified as a ceramide species, exhibited pronounced increased abundance in the Pfizer group (AUROC = 0.996, 95% CI: 0.99–1.00), contrasting with stable levels in AstraZeneca and Moderna groups. C_4_H_3_Cl_3_NS (**Figure 7C**), characterised as an acylcarnitine, displayed a distinct decrease in the Pfizer group (AUROC = 1.00), contrasting with stable or increasing trends in other vaccines. C_9_O_3_S_2_Esi+28.114012 (**Figure 7D**), identified as a phosphatidylcholine species, maintained consistently higher levels in Pfizer recipients across timepoints (AUROC = 1.00). These features therefore represent candidate Pfizer-associated molecular markers within this discovery cohort. Their biological significance, platform specificity, and discriminatory performance require confirmation in independent cohorts and using orthogonal analytical assays.

**Figure 7:**
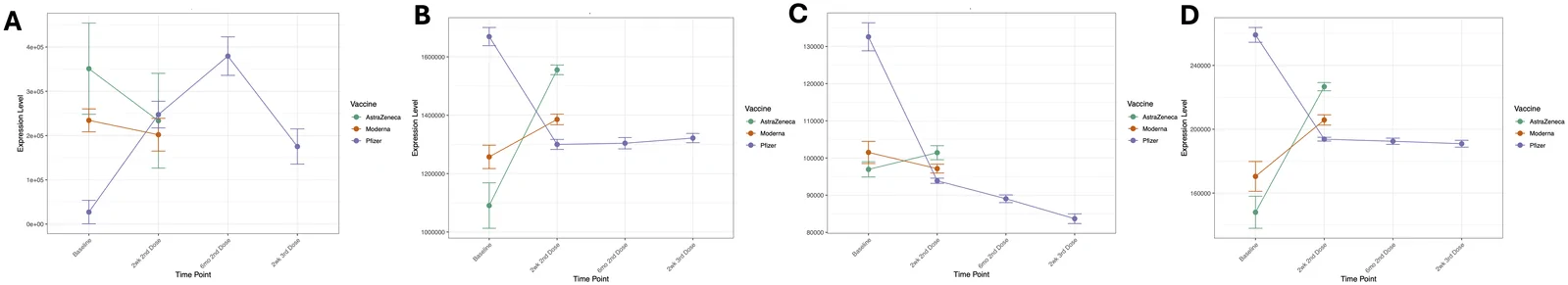
Time-course expression profiles of lipidomic candidate biomarkers uniquely associated with the Pfizer vaccine. **(A)** 1517.14@10.395997 **(B)** C_10_NS_2_ (ceramide species) **(C)** C_4_H_3_Cl_3_NS (acylcarnitine) **(D)** C_9_O_3_S_2_Esi+28.114012 (phosphatidylcholine species).

Metabolite candidate biomarkers comprised L-phenylalanine (13C-labeled) (**Figure 8A**) and citric acid (**Figure 8B**), both showing distinct temporal trajectories unique to Pfizer vaccination. L-phenylalanine 13C demonstrated sustained decrease only in Pfizer recipients, while levels remained unchanged in other vaccine groups. This metabolite achieved strong discriminatory performance, with an AUROC of 0.88 (95% CI: 0.77–1.00) between baseline and post-vaccination samples. Citric acid showed a progressive increase over time in Pfizer recipients, contrasting with marked decreases at equivalent timepoints in both AstraZeneca and Moderna recipients. Its classification performance was also robust (AUROC = 0.80, 95% CI: 0.66–0.94), further supporting its role as a potential Pfizer-specific metabolic candidate biomarker.

**Figure 8:**
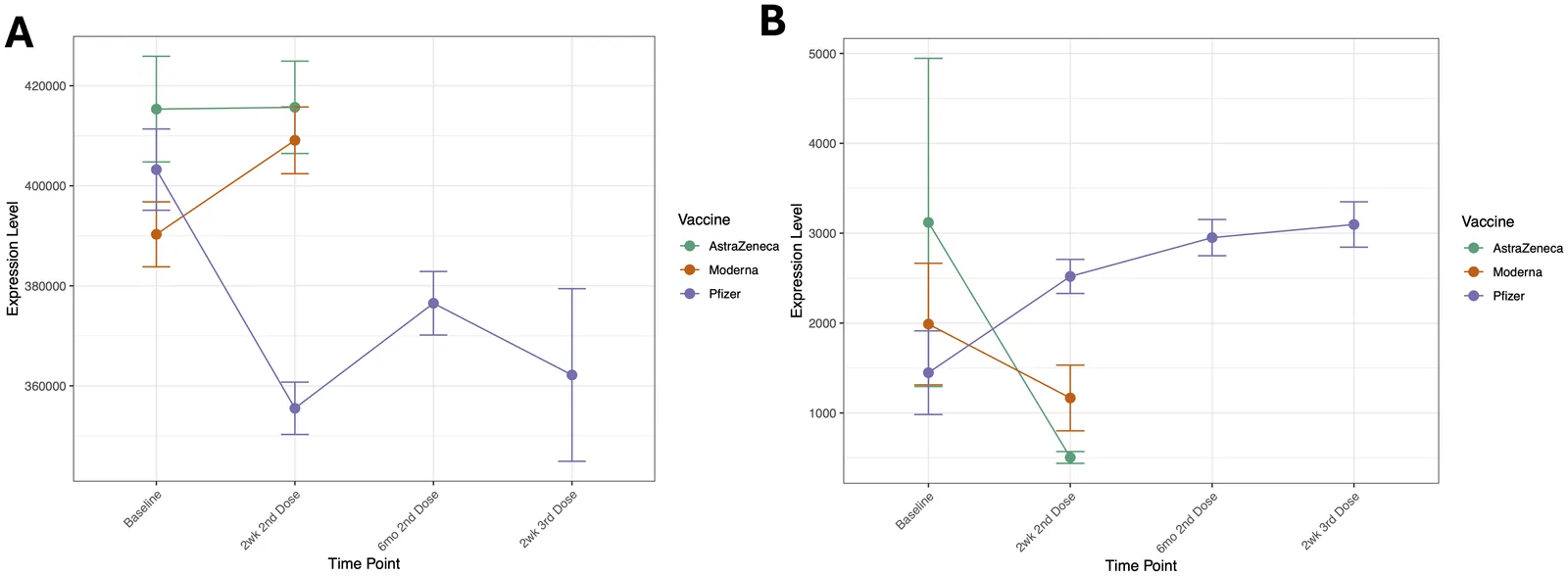
Time-course expression profiles of metabolomic candidate biomarkers uniquely associated with the Pfizer vaccine. **(A)** L-phenylalanine (13C-labeled) **(B)** citric acid.

Protein candidate biomarkers demonstrated the most diverse vaccine-specific signature, with five proteins showing unique regulation profiles in Pfizer-vaccinated individuals at two weeks post-second dose. Complement C5 (P01031.4) (**Figure 9A**) exhibited pronounced increased abundance in the Pfizer group, and achieved high within-cohort discriminatory performance (AUROC = 0.86, 95% CI: 0.74–0.98). Two immunoglobulin heavy variable proteins, IGHV4-28 (A0A0C4DH34.1) (**Figure 9B**) and IGHV5-51 (A0A0C4DH38.1) (**Figure 9C**), showed higher abundance in Pfizer recipients, demonstrating distinct abundance patterns among the selected immunoglobulin variable protein; their discriminatory capacity was moderate (AUROC = 0.66 and 0.80, respectively). Notably, U2 snRNP auxiliary factor 35 kDa subunit-related protein (Q15695.2) (**Figure 9D**) demonstrated strong vaccine-specific separation (AUROC = 0.87, 95% CI: 0.76–0.98) while IGHV3-64 (A0A075B6Q5.1) (**Figure 9E**) achieved moderate classification power (AUROC = 0.75, 95% CI: 0.61–0.90). Collectively, these proteins represent candidate Pfizer-associated molecular markers within the present cohort. Their reproducibility, platform specificity, and potential clinical utility require confirmation in independent cohorts and using orthogonal protein assays.

**Figure 9:**
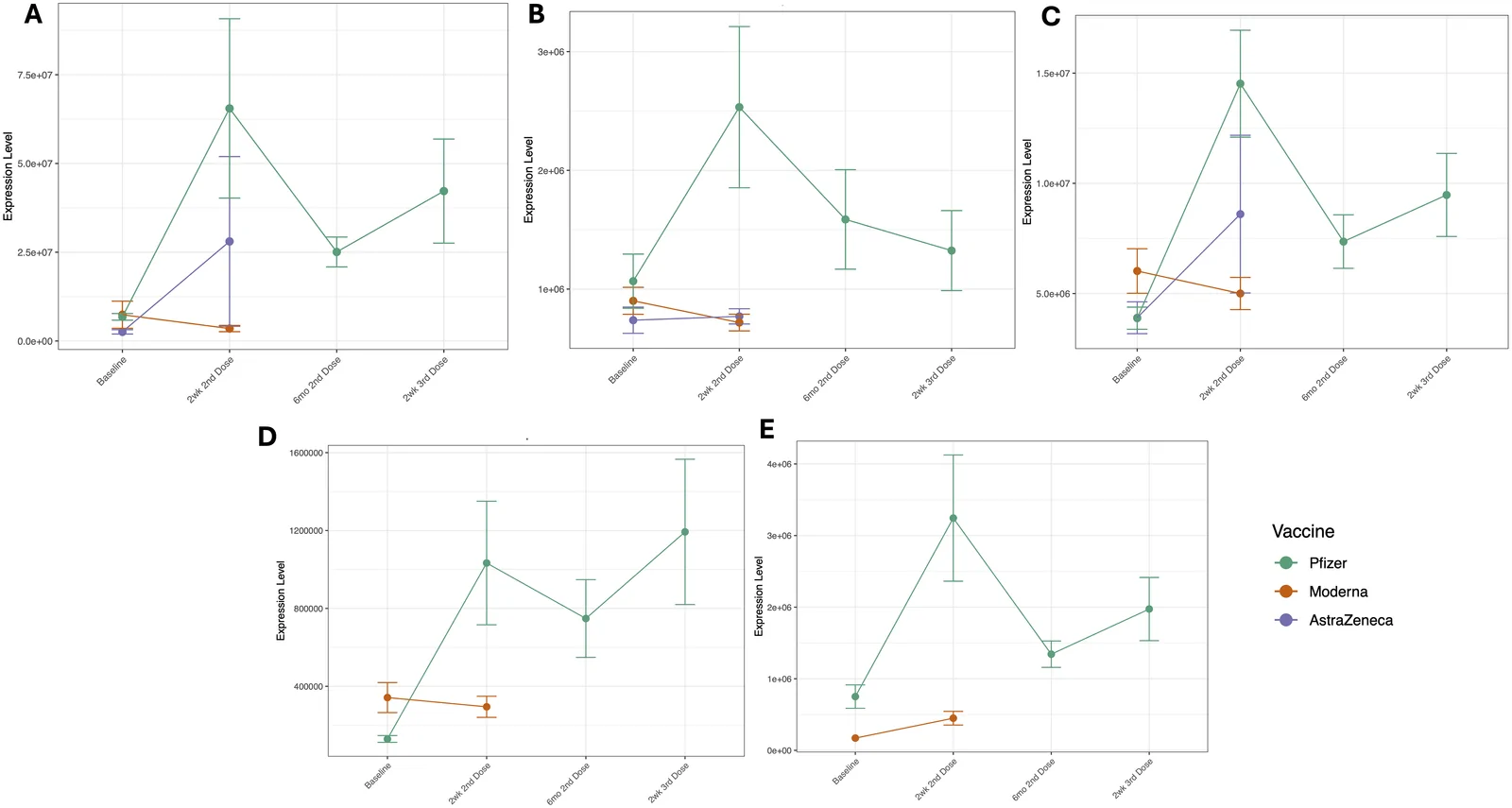
Time-course expression profiles of proteomic candidate biomarkers uniquely associated with the Pfizer vaccine. **(A)** Complement C5 (P01031.4) **(B)** IGHV4-28 (A0A0C4DH34.1) **(C)** IGHV5-51 (A0A0C4DH38.1) **(D)** U2 snRNP auxiliary factor 35 kDa subunit-related protein (Q15695.2) **(E)** IGHV3-64 (A0A075B6Q5.1).

## Discussion

This study provides a comprehensive, integrated multi-omics comparison of molecular responses to Pfizer-BioNTech, Moderna, and AstraZeneca COVID-19 vaccines. We show that vaccine platforms are associated with distinct multi-omics molecular signatures with different temporal patterns. By jointly analysing proteomic, lipidomic, and metabolomic layers, we uncover potential molecular signatures that are not apparent from single-omics profiling alone. Although all three vaccines encode the SARS-CoV-2 spike glycoprotein, the different platforms were associated with distinct systemic molecular response patterns.

### Divergent molecular signatures between mRNA vaccines

A central finding is the marked difference between Pfizer and Moderna vaccine responses at the protein, metabolic, and lipidomic levels. Pfizer vaccination was associated with progressively broader decrease in lipid abundance and predominantly increased polar metabolites abundance at later timepoints. In contrast, Moderna vaccination showed a predominantly increased lipidomic profile together with bidirectional metabolite changes and minimal univariate proteomic changes. These observations define distinct molecular response patterns but do not by themselves establish different metabolic programs or mechanisms.

These distinct signatures emerged despite both vaccines being mRNA-based and sharing similar antigen targets. Differences in mRNA dose, lipid nanoparticle composition, administration interval, or other platform- and cohort-associated factors could contribute to the observed patterns, although the present study was not designed to determine their causal contribution. Although prior studies have reported differences in antibody magnitude and Fc effector function between Pfizer and Moderna (36, 43), the present data therefore extends these comparisons by identifying opposite-direction lipid abundance patterns between the two cohorts. Replication in independent cohorts will be required to determine the reproducibility and biological basis of these differences.

### Integrated proteomic and metabolic patterns in Pfizer responses

Pfizer responses were distinguished not only by their breadth but also by their temporal orchestration across molecular layers. Proteomic analysis showed broad increases in immunoglobulin and complement-associated proteins at 2wk-2nd, fewer differentially abundant proteins at 6mo-2nd, and renewed increases in selected complement-associated proteins after booster dose. These abundance patterns are compatible with known temporal immune responses to vaccination, but they do not directly measure B-cell expansion, complement activity, or immune memory. These findings are consistent with systems vaccinology studies of BNT162b2, which reported robust innate transcriptional activation and strong adaptive responses (36). Complement and acute-phase signatures after mRNA boosting have also been documented in high-throughput proteomic studies (43). *In vitro* studies have shown that lipid-based nanoparticle formulations can activate the complement system, providing a plausible biological context for the complement-associated abundance changes observed here (44).

In parallel, lipidomic analysis revealed no evidence of resolution; instead, suppression intensified across timepoints, with the greatest number of significantly altered lipids observed after the third dose. Metabolomic profiling revealed a temporal shift from an initially bidirectional response at 2wk-2nd to sustained net upregulation at later stages, particularly of amino acid derivatives and sugar intermediates. This late-stage pattern raises the possibility of persistent metabolic changes following vaccination; however, plasma metabolite abundance alone cannot establish metabolic imprinting or its relationship to vaccine-response longevity. While antibody waning and recall over six months have been well described (45), long-term trajectories of lipid and metabolic systems have not previously been reported. The proteomic abundance patterns measured here do not themselves establish immune memory. Lipidomic and metabolomic abundance patterns remained altered at later sampled timepoints, although their functional significance and relationship to longer-term vaccine responses require further investigation.

### Moderna responses emphasise metabolic–lipid coordination

In contrast, Moderna responses were characterised by moderate but consistent inter-omics coordination, particularly between lipidomic and metabolomic compartments. Increased abundance of acylcarnitines, amino acids and nucleotide-related metabolites could be consistent with changes in mitochondrial lipid metabolism, biosynthetic activity, or nucleotide metabolism; however, mitochondrial oxidation, protein synthesis, cellular proliferation, and immune-cell expansion were not directly measured. The relatively limited univariate proteomic changes indicate that the strongest measured Moderna-associated changes occurred in the lipidomic and metabolomic layers.

Although direct metabolomic studies of mRNA-1273 remain sparse, existing proteomic and serologic assessments in BNT162b2 (Pfizer-BioNTech) recipients confirm complement and metabolic pathway involvement (46). More broadly, comparative analyses of Pfizer and Moderna vaccine recipients have shown higher antibody titers and enhanced Fc effector functions after Moderna (47), providing broader immunological context for the molecular differences observed here. The present study extends these findings by providing, to our knowledge, one of the first integrated lipid–metabolite multi-omics analyses of COVID-19 vaccine responses, identifying correlated lipid–metabolite signatures associated with Moderna vaccination.

### Subtle but coordinated responses to AstraZeneca

Although conventional DE analyses revealed no significant proteins and only a limited number of lipids following AstraZeneca vaccination, multi-omics integration identified a subtle but coordinated cross-omic molecular signature. The discriminant features included lipid, amino-acid, and complement-associated molecules; however, DIABLO-derived associations do not establish regulation of these pathways. Modest changes in several metabolites, including glutamine and TCA-cycle-associated intermediates, were also observed. Given the small AstraZeneca cohort (n = 7), these findings should be considered exploratory and require confirmation in larger cohorts.

## Conclusion

In summary, this study demonstrates that COVID-19 vaccines are associated with platform-specific and temporally distinct molecular responses, showing coordinated multi-omics signatures that extend far beyond antibody production. By uncovering markedly different lipidomic and metabolomic response patterns between Pfizer and Moderna, identifying subtle but coherent AstraZeneca response, and identifying candidate platform-specific biomarkers, our findings provide a foundation for future precision vaccinology studies. Multi-omics integration thus provides not only a deeper molecular characterisation of vaccine responses but also a potential framework for informing future studies of vaccine development and personalised vaccination strategies. These molecular differences should not be interpreted as evidence of distinct biological programs or mechanisms, which will require functional validation.

### Broader implications for vaccinology

Our findings underscore the value of multi-omics integration in decoding complex vaccine-induced biology. Prior systems vaccinology has revealed differences in antibody functionality (47) and platform-specific innate trajectories (43, 48), but our data show that vaccines are also associated with distinct long-term molecular profiles. The demonstration that two mRNA vaccines with similar delivery technologies show opposing lipidomic abundance patterns highlights the need to move beyond antigen-focused models of vaccine action and consider the systemic biological context in which immunity is generated. These findings identify metabolic and lipid pathways that warrant further investigation in vaccine development studies. Furthermore, the persistence of lipidomic and metabolomic abundance changes across the sampled Pfizer timepoints motivates further study of the longer-term molecular effects of repeated vaccination.

### Limitations and future directions

This study provides an initial integrated multi-omics characterisation of systemic molecular responses to three widely used COVID-19 vaccine platforms and establishes a foundation for subsequent validation and mechanistic studies. The smaller AstraZeneca cohort (n = 7) warrants cautious interpretation of platform-specific findings, particularly multivariate classification results, and larger independent cohorts will be important to confirm these signatures. Although age and sex were included in sensitivity analyses, residual demographic confounding cannot be completely excluded. The study focused on plasma rather than paired cellular profiling. Plasma is a molecularly rich and clinically accessible biofluid that captures circulating proteins, lipids, and metabolites and is therefore well suited to identifying systemic vaccine-associated molecular signatures. Similarly, the absence of clinical efficacy endpoints reflects the primary objective of this study, which was to characterise vaccine-associated molecular responses rather than evaluate vaccine effectiveness or clinical outcomes. Future studies integrating these molecular profiles with immunological and clinical endpoints will be important for determining their relationship to protection, durability, and adverse-event profiles. Candidate molecular markers identified here should therefore be viewed as discovery-stage candidates, requiring confirmation in independent cohorts and orthogonal analytical assays. Finally, further structural characterisation of currently unannotated lipid and metabolite features and extended longitudinal sampling across all vaccine platforms will help define the biological relevance and durability of the molecular signatures identified here.

## Data Availability

The datasets presented in this study are publicly available in the CSIRO Data Access Portal at: https://data.csiro.au/collection/csiro:77992.
