# Supplementary material for: Multi-omics integration reveals vaccine-specific molecular signatures and temporal dynamics of COVID-19 immune responses
Source: Front Immunol. 2026 Oct 2;17:1815543. doi: 10.3389/fimmu.2026.1815543 (PMC13672565; doi:10.3389/fimmu.2026.1815543)
Supplement: Supplementary file 1 [file Supplementaryfile1.docx]

**Supplementary Methods**

Within each (vaccine × omics) subset, features were filtered using omics-specific missingness thresholds. Features with >40%, >35%, or >30% missing values overall were excluded for proteomics, lipidomics, and metabolomics, respectively. Features were additionally required to have ≤60% missing values within each pre- and post-vaccination group for proteomics and lipidomics and ≤50% for metabolomics. For lipidomics, vendor-encoded values of exactly 1 were treated as missing prior to filtering. After normalisation, any remaining NA values were mean-imputed per feature only for the PCA step. Prior to PCA, features with zero variance were removed (variance > 0 criterion).

**Baseline comparability across vaccine platforms**

**Supplementary Figure 1. Baseline comparability of molecular profiles across vaccine platforms following normalisation.** Principal component analysis (PCA) plots of baseline plasma samples for (A) lipidomics, (B) metabolomics, and (C) proteomics reveal no distinct clustering by vaccine platform (Pfizer-BioNTech, Moderna, AstraZeneca) after platform-specific normalisation. Overlapping 95% confidence ellipses indicate similar global molecular profiles at baseline across all vaccine groups, confirming the absence of systematic pre-vaccination biases. These results support the validity of downstream comparisons and indicate that observed post-vaccination differences are unlikely to be driven by artefacts or baseline disparities.

**Normalisation pipelines**

We benchmarked six pipelines, each applied column-wise (per feature across samples). All steps below are performed in the order listed.

1. **Default**:
   (i) min/3 imputation: each missing entry is set to one third of the feature’s observed minimum;
   (ii) min–max normalisation: $x^{'}=(x-\min)/(\max-\min)$computed per feature;
   (iii) Pareto scaling: mean-center each feature and divide by $\sqrt{\mathrm{SD}}$.
2. **Quantile_zscore**:
   (i) “KNN-like” placeholder imputation implemented as feature mean imputation (i.e., missing values replaced by the feature’s mean of non-missing entries);
   (ii) quantile normalisation implemented as rank transformation to the unit interval $[0,1]$(value = average rank / length);
   (iii) z-score scaling per feature.
3. **Log2_Autoscale**:
   (i) feature mean imputation;
   (ii) $\log_{2}(x+1)$transform;
   (iii) z-score scaling per feature.
4. **Complete_pipeline**:
   (i) min/3 imputation;
   (ii) $\log_{10}(x+0.001)$transform;
   (iii) Pareto scaling;
   (iv) z-score scaling.
5. **VSN_pipeline**:
   (i) min/3 imputation;
   (ii) variance-stabilising normalisation (VSN; vsn2) applied to the transposed matrix (feature-wise), with an automatic positive offset if needed; if the data matrix was too small (n < 3 samples or < 2 features) or VSN failed, fallback to $\log_{2}(x+1)$.
6. **Loess_pipeline**:
   (i) feature mean imputation;
   (ii) cyclic loess normalisation (limma::normalizeCyclicLoess); for matrices with <3 samples or on error, fallback to $\log_{2}(x+1)$;
   (iii) z-score scaling per feature.

**Principal component analysis (PCA)**

For each (vaccine × omics × pipeline) normalised matrix, PCA was computed with prcomp(center = TRUE, scale. = TRUE). Before PCA only, any residual missing entries were replaced by the feature mean, and zero-variance features were removed as described above. Percent variance explained by PC1 and PC2 was extracted from the PCA importance table.

**Timepoint separation metric**

To quantify longitudinal structure, we computed a timepoint separation score on the first two PCs. For each timepoint, we calculated the centroid in (PC1, PC2) space and then took the mean of all pairwise Euclidean distances between centroids. Larger values indicate greater separation of timepoints along the primary PCA axes.

**Pipeline ranking**

Within each (vaccine × omics) context, pipelines that completed successfully were ranked using a composite score:

$$\text{Score}=0.4\times\mathrm{scale}(\text{PC1 variance \%})+0.6\times\mathrm{scale}(\text{timepoint separation})$$

where scale denotes standardisation across pipelines within the same (vaccine × omics) set. The highest composite score defined the top-performing pipeline. We also report the number of features retained after variance filtering used by the PCA.

**AstraZeneca lipidomics: pipeline comparison**

We next compared the six normalisation pipelines on the AstraZeneca lipidomics dataset (**Supplementary Fig. S2**). All pipelines produced stable PCA structures, but the Default pipeline achieved the strongest balance of variance capture and timepoint separation (PC1 = 28.9%, PC2=16.6%, separation = 19.05). These findings indicate that while multiple pipelines are feasible, the Default approach maximises signal-to-noise resolution in this dataset.

**Supplementary Figure 2.** PCA comparison of normalisation pipelines (AstraZeneca lipidomics).

**AstraZeneca lipidomics: distributional and QC analyses**

**Supplementary Figure 3.** Log2_Autoscale diagnostic analysis (AstraZeneca lipidomics)

**AstraZeneca metabolomics: pipeline comparison**

Six normalisation pipelines were evaluated on the AstraZeneca metabolomics dataset (**Supplementary Fig. S4**). The **Loess_pipeline** achieved the highest apparent variance capture (PC1 = 73.4%), but further assessments showed that it **oversmooths the metabolomics data and collapses true biological differences**, leading to **poor timepoint separation** despite high variance. In contrast, the **Complete pipeline** produced a more balanced structure (PC1 = 42%, PC2 = 9.7%) while maintaining strong longitudinal separation (separation = 25.65). The **Default** pipeline also achieved high separation (26.34) but at the expense of lower explained variance (PC1 = 39%). Based on the balance of variance retention and separation, and to avoid overfitting artefacts, the Default pipeline was selected as the most appropriate normalisation for this dataset.

**Supplementary Figure 4.** PCA comparison of six normalisation pipelines for AstraZeneca metabolomics. Scatterplots show PC1 vs. PC2 projections with samples coloured by timepoint (baseline, post-vaccination). Barplots summarise PC1/PC2 variance explained and mean separation between timepoint centroids.

**AstraZeneca metabolomics: Distributional and QC analyses**

To evaluate data structure and assess the impact of normalisation on the AstraZeneca metabolomics dataset, we inspected feature distributions and sample–sample correlation matrices (**Supplementary Fig. S5**). The raw intensity values exhibited strong right-skew and large between-sample variability, with several samples showing markedly higher overall signal. Correspondingly, the raw correlation heatmap displayed heterogeneous pairwise similarity, indicating that technical variation dominated over biological structure (bottom-left panel; Following transformation using the **Complete** pipeline, the metabolite distributions were substantially stabilised, with signals compressed into a narrow and more symmetric range across the 14 samples. This normalisation reduced intensity-driven artefacts and minimised the influence of extreme values. The normalised correlation matrix showed a clearer diagonal structure, reflecting increased internal consistency across samples and improved comparability between baseline and 2-week post–dose measurements. Overall, the Complete pipeline markedly reduced technical noise and harmonised metabolite profiles within the AstraZeneca cohort, making it suitable for downstream PCA-based evaluation and group-level comparisons.

**Supplementary Figure S5.** Diagnostic evaluation of the Complete pipeline applied to AstraZeneca metabolomics. Density distributions and PCA plots of raw, normalised, and scaled data illustrate improved distributional properties and robust longitudinal separation.

**AstraZeneca proteomics: pipeline comparison**

Six normalisation pipelines were evaluated on the AstraZeneca proteomics dataset (14 samples; baseline and 2 weeks post–second dose) (**Supplementary Fig. S6**). The **Loess_pipeline** appeared to achieve the strongest performance, capturing nearly all variance on PC1 (97.4%) with the highest longitudinal separation (8.85). However, this variance structure was dominated by a single axis, indicating overfitting and limiting biological interpretability. By contrast, the **Default pipeline** (min/3 imputation → min–max normalization → Pareto scaling) provided a more balanced representation (PC1 = 51.5%, PC2 = 19.2%) while retaining strong timepoint separation (7.22). For this reason, the Default pipeline was selected as the most appropriate approach, as it preserves proteomic structure without overfitting.

**Supplementary Figure S6.** PCA comparison of six normalisation pipelines for AstraZeneca proteomics. Scatterplots show PC1 vs. PC2 projections with samples colored by timepoint. Barplots summarise PC1/PC2 variance explained and mean separation between timepoint centroids.

**AstraZeneca proteomics: Distributional and QC analyses**

A detailed evaluation of the **Default pipeline** confirmed these improvements (**Supplementary Fig. S7**). Density plots showed compression of extreme values and stabilisation of feature-level distributions, while PCA demonstrated preserved major variance (PC1 = 51.5%, PC2 = 19.2%) with clear separation between baseline and post-vaccination groups (separation = 7.22).

**Supplementary Figure S7.** Raw versus normalised feature distributions and correlation matrices for AstraZeneca proteomics. Normalisation improved feature symmetry and enhanced sample comparability.

Raw proteomic intensities displayed skewed feature distributions and heterogeneous sample scaling (**Supplementary Fig. S8**). Normalisation improved distributional symmetry and increased inter-sample correlations, indicating more consistent structure across baseline and post-vaccination samples.

**Supplementary Figure S8.** Diagnostic evaluation of the Default pipeline applied to AstraZeneca proteomics. Density distributions and PCA plots of raw, normalised, and scaled data illustrate improved variance structure and clear longitudinal separation.

**Moderna lipidomics: pipeline comparison**

We benchmarked six normalisation pipelines on the Moderna lipidomics dataset (48 samples; baseline and 2 weeks post–second dose) (**Supplementary Fig. S9**). The **Loess_pipeline** appeared to yield the strongest performance, capturing the majority of variance (PC1 = 74.8%) and achieving the highest longitudinal separation (25.38). However, this extreme dominance of PC1 indicated overfitting, limiting interpretability. In contrast, the **Quantile_zscore** pipeline produced a more balanced variance structure (PC1 = 25.1%, PC2 = 9%) while still achieving strong timepoint discrimination (separation = 17.33). For this reason, the Quantile_zscore method was selected as the most appropriate normalisation, providing robust longitudinal resolution without overfitting.

**Supplementary Figure S9.** PCA comparison of six normalisation pipelines for Moderna lipidomics. Scatterplots show PC1 vs. PC2 projections with samples coloured by timepoint (baseline, post-vaccination). Barplots summarise PC1/PC2 variance explained and mean separation between timepoint centroids. Loess_pipeline captured the most variance but was overfit; Quantile_zscore provided the most interpretable balance of variance retention and separation.

**Moderna lipidomics distributional and QC analyses**

Raw lipidomics data displayed strong skew and heterogeneity across features. After normalisation with the Quantile_zscore pipeline, feature distributions became more symmetrical and inter-sample correlations increased (**Supplementary Fig. S10**). These improvements highlight enhanced comparability across baseline and post-vaccination samples.

**Supplementary Figure S10.** Diagnostic evaluation of the Quantile_zscore pipeline applied to Moderna lipidomics. Density distributions and PCA plots of raw, normalised, and scaled data illustrate improved feature scaling and enhanced longitudinal separation.

**Moderna metabolomics: pipeline comparison**

Six normalisation pipelines were assessed on the Moderna metabolomics dataset (48 samples; baseline and 2 weeks post–second dose) (**Supplementary Fig. S11**). Although the Loess_pipeline yielded the highest variance explained (PC1 = 71.5%), this came at the cost of unstable structure and poor longitudinal discrimination, consistent with overfitting. In contrast, the Quantile Z-score pipeline provided the **best overall balance**, capturing a moderate proportion of variance (PC1 = 27.7%, PC2 = 5.2%) while also achieving **the highest biologically meaningful separation between baseline and post-vaccination samples** (separation = 21.76). Importantly, Quantile Z-score preserved the natural sample relationships without inducing artificial compression of baseline values, as observed in the Loess method. For these reasons, despite not having the largest PC1, the Quantile Z-score **was selected as the optimal normalisation approach for the Moderna metabolomics dataset**, offering the most stable and interpretable structure for downstream analyses.

**Supplementary Figure S11.** PCA comparison of six normalisation pipelines for Moderna metabolomics. Scatterplots show PC1 vs. PC2 projections with samples coloured by timepoint. Barplots summarise PC1/PC2 variance explained and mean separation between timepoint centroids.

**Moderna metabolomics distributional and QC analyses**

Raw metabolite intensities exhibited substantial skew and inconsistent scaling across samples, as visible from the wide and heterogeneous raw boxplots (**Supplementary Fig. S12, top left**). After applying the Quantile Z-score, feature distributions became markedly more uniform, with compressed interquartile ranges and improved symmetry (**Supplementary Fig. S12, top right**). Correspondingly, the raw correlation matrix showed strong diagonal structure but high variability across off-diagonal correlations, indicating batch or intensity-driven artefacts (**Supplementary Fig. S12, bottom left**). Normalisation substantially reduced this heterogeneity, producing a more homogeneous correlation landscape with clearer sample–sample consistency (**Supplementary Fig. S12, bottom right**). Together, these changes confirm that the Quantile Z-score pipeline effectively stabilised variance, harmonised sample scaling, and improved comparability across Moderna metabolomics profiles.

**Supplementary Figure S12.** Diagnostic evaluation of the Complete pipeline applied to Moderna metabolomics. Density distributions and PCA plots of raw, normalised, and scaled data illustrate improved variance stabilisation and enhanced separation between timepoints.

**Moderna proteomics: pipeline comparison**

Six normalisation pipelines were evaluated on the Moderna proteomics dataset (48 samples; baseline and 2 weeks post–second dose) (**Supplementary Fig. S13**). The **Loess_pipeline** appeared to capture the majority of variance (PC1 = 90.2%) and yielded the strongest apparent separation (5.86). However, this was indicative of overfitting, as nearly all variance was compressed into a single component, limiting biological interpretability. By contrast, the **Default pipeline** provided a more balanced representation of the data (PC1 = 37.1%, PC2 = 14.9%, separation = 2.74), preserving proteomic structure while still differentiating baseline from post-vaccination samples. For this reason, the Default pipeline was selected as the most appropriate normalisation strategy for Moderna proteomics.

**Supplementary Figure S13.** PCA comparison of six normalisation pipelines for Moderna proteomics. Scatterplots show PC1 vs. PC2 projections with samples coloured by timepoint. Barplots summarise PC1/PC2 variance explained and mean separation between timepoint centroids. Loess_pipeline maximised variance capture but was considered overfitted, whereas the Default pipeline provided the most stable and interpretable balance.

**Moderna proteomics distributional and QC analyses**

A focused evaluation of the Default pipeline confirmed its effectiveness (**Supplementary Fig. S14**). Density plots showed stabilization of feature-level distributions after min/3 imputation, min–max normalisation, and Pareto scaling. PCA on raw, normalised, and final scaled data demonstrated preserved major variance (PC1 = 37.1%, PC2 = 14.9%) with improved separation between baseline and post-vaccination samples (separation = 2.74).

**Supplementary Figure S14.** Raw versus normalised feature distributions and correlation matrices for Moderna proteomics. Normalisation improved distributional symmetry and increased inter-sample consistency across the 48 samples.

Raw proteomic intensity distributions were skewed and showed heterogeneity across samples. After normalisation, feature-level symmetry improved and inter-sample correlations increased (**Supplementary Fig. S15**). This normalisation effect enhanced overall comparability between baseline and post-vaccination groups.

**Supplementary Figure S15.** Diagnostic evaluation of the Default pipeline applied to Moderna proteomics. Density distributions and PCA plots of raw, normalised, and scaled data illustrate improved variance stabilisation and modest longitudinal separation.

**Pfizer lipidomics: pipeline comparison**

Six normalisation pipelines were benchmarked on the Pfizer lipidomics dataset (75 samples across baseline, 2 weeks after second dose, 6 months after second dose, and 2 weeks after third dose) (**Supplementary Fig. S16**). The Loess_pipeline produced the largest apparent variance structure (PC1 = 79.2%) and the highest nominal separation (31.6). However, inspection of the corresponding boxplots and correlation matrices revealed clear evidence of overfitting, with excessively compressed distributions and artificially inflated sample–sample similarity. In contrast, the VSN_pipeline underperformed, retaining limited variance (PC1 = 26.3%) and providing minimal longitudinal discrimination (15.75), consistent with an over-regularised transformation. Among the remaining methods, Default, Quantile_zscore, Log2_Autoscale, and the Complete_pipeline all produced stable variance profiles (PC1 ≈ 39–44%; PC2 ≈ 9.7–10.4%) and comparable separation metrics (~27–29). The Complete_pipeline offered the strongest overall balance, achieving the highest biologically plausible variance capture (PC1 = 44.3%, PC2 = 10.1%) and the greatest non-overfitted timepoint separation (28.86).

**Supplementary Figure S16.** PCA comparison of six normalisation pipelines for Pfizer lipidomics. Scatterplots show PC1 vs. PC2 projections with samples coloured by timepoint. Barplots summarise PC1/PC2 variance explained and mean separation between timepoint centroids..

**Pfizer lipidomics distributional and QC analyses**

The QC plots further supported this choice: normalised distributions were symmetrical without distortion, and correlation structure improved without collapsing samples into an artefactual cluster (**Supplementary Fig. S17**). For these reasons, the Complete_pipeline was selected as the preferred normalisation approach for Pfizer lipidomics, as it maximised biological interpretability while avoiding both over- and underfitting.

**Supplementary Figure S17.** Diagnostic evaluation of the Log2_Autoscale pipeline applied to Pfizer lipidomics. Density distributions and PCA plots of raw, normalised, and scaled data illustrate improved variance stabilisation and enhanced separation between baseline and multiple post-vaccination timepoints.

**Pfizer metabolomics: pipeline comparison**

We benchmarked six normalisation pipelines on the Pfizer metabolomics dataset (75 samples; baseline, 2 weeks after second dose, 6 months after second dose, and 2 weeks after third dose) (**Supplementary Fig. S18**). The **Loess_pipeline** appeared to achieve the strongest performance, capturing the majority of variance (PC1 = 76.2%). However, this dominance of a single axis indicated overfitting and limited interpretability. In contrast, the **Quantile Z-score pipeline** produced a more balanced outcome, explaining 24.5% of PC1 variance with a separation score of 16.44. For this reason, the **Quantile Z-score** pipeline was selected as the most appropriate normalisation for Pfizer metabolomics, as it retained meaningful biological structure without overfitting.

**Supplementary Figure S18.** PCA comparison of six normalisation pipelines for Pfizer metabolomics. Scatterplots show PC1 vs. PC2 projections coloured by timepoint. Barplots summarise PC1/PC2 variance explained and mean timepoint separation.

**Pfizer metabolomics distributional and QC analyses**

A focused evaluation of the **Quantile Z-score** confirmed its suitability. Raw metabolite intensities showed skewed distributions and heterogeneous sample scaling. After normalisation with the **Quantile Z-score**, feature distributions became more symmetrical and sample–sample correlations increased (**Supplementary Fig. S19**). These improvements enhanced data comparability across the 75 Pfizer metabolomics samples.

**Supplementary Figure S19.** Diagnostic evaluation of the **Quantile Z-score** applied to Pfizer metabolomics. Density distributions and PCA plots of raw, normalised, and scaled data demonstrate effective variance stabilisation and enhanced longitudinal discrimination.

**Pfizer proteomics: pipeline comparison**

We evaluated six normalisation pipelines on the Pfizer proteomics dataset (75 samples; baseline, 2 weeks after second dose, 6 months after second dose, and 2 weeks after third dose) (**Supplementary Fig. S20**). The **Loess_pipeline** appeared to achieve the strongest performance, explaining 95.7% of variance in PC1 and yielding the greatest longitudinal separation (7.50). However, this extreme concentration of variance into a single principal component was indicative of overfitting and reduced biological interpretability. In contrast, the **Complete_pipeline** (min/3 imputation → log10 transform → Pareto scaling → z-score) provided a more balanced outcome (PC1 = 56.4%, PC2 = 8.3%, separation = 7.11), making it better suited for downstream interpretation. For this reason, the Complete_pipeline was selected as the most appropriate normalisation strategy for Pfizer proteomics.

**Supplementary Figure S20.** PCA comparison of six normalisation pipelines for Pfizer proteomics. Scatterplots show PC1 vs. PC2 projections coloured by timepoint. Barplots summarise PC1/PC2 variance explained and mean separation between timepoint centroids. Although Loess_pipeline maximised variance, it was overfitted; the Complete_pipeline provided the most balanced and interpretable structure.

**Pfizer proteomics distributional and QC analyses**

A focused evaluation of the Complete_pipeline confirmed its utility (**Supplementary Fig. S21**). Density plots showed that log10 transformation compressed extreme values, while Pareto scaling and z-score normalisation stabilised feature variance. PCA of raw, normalised, and scaled data demonstrated preserved structure (PC1 = 56.4%, PC2 = 8.3%) with improved discrimination between baseline and post-vaccination samples (separation = 7.11).

**Supplementary Figure S21.** Raw versus normalised feature distributions and sample correlation matrices for Pfizer proteomics. Normalisation reduced skew and improved inter-sample consistency across all timepoints.

Raw proteomic intensities displayed marked skew and variable scaling across features. Normalisation improved feature distributions and strengthened inter-sample correlations (**Supplementary Fig. S22**), enhancing data comparability across the 75 Pfizer proteomics samples.

**Supplementary Figure S22.** Diagnostic evaluation of the Complete_pipeline applied to Pfizer proteomics. Density distributions and PCA plots of raw, normalised, and scaled data illustrate effective variance stabilisation and enhanced longitudinal separation.

**Differential expression analysis for AstraZeneca**

**Supplementary Figure S23.** Differential expression analysis across omics layers following AstraZeneca vaccination. (**A**) Metabolomics layer,(**B**) Lipidomics layer, and (**C**) Proteomics layer.

**Supplementary Figure S24. Demographic comparison of participants across vaccine platforms.** (A) Sex distribution (F/M) by vaccine group shows no significant difference (Fisher's exact test, P = 0.4818). (B) Year of birth by vaccine group shows a statistically significant difference (ANOVA, P = 0.0222), with the AstraZeneca group comprising older individuals on average.

**Robustness to alternative preprocessing and normalisation**

To assess whether the principal differential-abundance findings depended on the selected preprocessing strategy, each vaccine–omics comparison was repeated using alternative normalisation pipelines while retaining identical samples, missingness filters, covariates, statistical models, and multiple-testing procedures. The principal molecular response patterns were generally robust across preprocessing approaches. Pfizer lipidomic effects showed strong concordance across alternative pipelines at all timepoints (Spearman $r_{s}=0.91\text{–}0.97$; direction concordance 96–99%), with substantial overlap of FDR-significant features (Jaccard similarity 0.85–0.96). Pfizer metabolomic effects were similarly stable ($r_{s}=0.98\text{–}0.99$; direction concordance 94–96%; Jaccard 0.85–0.90). Moderna and AstraZeneca metabolomic responses also showed high concordance across preprocessing approaches ($r_{s}\geq0.98$ for most comparisons), and the absence of significant proteomic changes following Moderna or AstraZeneca vaccination was reproduced across all evaluated pipelines.

Greater preprocessing dependence was observed for the number and identity of significant Moderna and AstraZeneca lipid features and for later Pfizer proteomic responses. Nevertheless, the predominant direction of the Moderna lipidomic response remained consistent across all pipelines, with increased features outnumbering decreased features under each preprocessing strategy. AstraZeneca lipidomic significance was less stable, supporting cautious interpretation of this small-cohort result. Overall, these analyses indicate that the principal directional and cross-omic conclusions were not driven by a single preprocessing approach, although exact feature-level significance calls were more sensitive to normalisation in selected datasets.

**Supplementary Figure S25:** **Robustness of differential-expression results to alternative preprocessing and normalisation strategies.** Differential-expression analyses were repeated using alternative preprocessing pipelines while maintaining identical sample inclusion, feature filtering, covariate adjustment, statistical modelling, and multiple-testing procedures. (**A**) Proportion of features retaining the same direction of effect between the primary and alternative analyses. (**B**) Spearman correlations between model-derived effect estimates obtained using the primary and alternative preprocessing pipelines. (**C**) Jaccard similarity of feature sets meeting an FDR threshold of <0.05 under the primary and alternative preprocessing strategies. High effect-estimate correlations and directional concordance indicate that the principal molecular response patterns were generally robust to preprocessing choice, whereas lower Jaccard similarity in selected datasets indicates greater sensitivity of exact feature-level significance calls to normalisation.

**DIABLO model validation and feature-set stability**

The prespecified DIABLO models comprised two latent components, with five variables retained from each omics block per component. These full-cohort models were used to generate the loading plots, sample projections, cross-omics correlations, network visualisations, and integrated heatmaps presented in the main figures. Model performance was evaluated using participant-grouped cross-validation, ensuring that all samples from the same participant were retained within the same training or test partition. Repeated grouped five-fold cross-validation was applied to the Pfizer-BioNTech and Moderna comparisons. Leave-one-participant-out cross-validation was used for the AstraZeneca comparison because of the smaller cohort size. Predictions generated for held-out participants were used to calculate balanced error rate, classification accuracy, and area under the receiver-operating-characteristic curve. To determine whether the observed discrimination exceeded that expected by chance, baseline and post-vaccination labels were permuted at the participant level while preserving the grouping of repeated samples. The complete grouped cross-validation procedure was repeated for each permutation. Empirical P-values were calculated from the proportion of permuted models achieving a balanced error rate equal to or lower than that of the observed model. Sensitivity to model specification was assessed using nested cross-validation, in which the number of latent components and the number of variables retained from each omics block were tuned within the training data. Performance of the nested-cross-validation-tuned models was compared with that of the prespecified DIABLO models. Potential model optimism was evaluated by comparing apparent accuracy obtained from the full fitted dataset with accuracy obtained from participant-grouped held-out predictions. Feature-set stability was assessed separately for the lipidomic, metabolomic, and proteomic blocks by calculating the mean pairwise Jaccard similarity between feature sets selected across participant resampling iterations. Jaccard similarity ranges from 0, indicating no overlap between selected feature sets, to 1, indicating identical feature sets.

**Supplementary Figure S26. Internal validation and feature-set stability of the prespecified DIABLO models (A)** Balanced error rate obtained using participant-grouped cross-validation for each vaccine and post-vaccination timepoint. The dashed horizontal line indicates chance-level performance at a balanced error rate of 0.5. Lower values indicate better classification. **(B)** Out-of-fold area under the receiver-operating-characteristic curve calculated from predictions for held-out participants. Error bars show the reported uncertainty intervals where available. **(C)** Participant-wise label-permutation analysis. Filled circles indicate the observed cross-validated balanced error rate, whereas open circles indicate the mean balanced error rate obtained after permutation of the baseline and post-vaccination labels. Error bars represent ±1 standard deviation of the permutation distribution. Empirical permutation P-values are shown above each comparison. **(D)** Comparison of the balanced error rate obtained using the prespecified DIABLO models and models tuned within nested cross-validation. Lines connect results from the same vaccine and timepoint comparison. **(E)** Comparison of apparent full-cohort classification accuracy and participant-grouped cross-validated accuracy. Small differences between the two estimates indicate limited model optimism. **(F)** Mean pairwise Jaccard similarity of the feature sets selected across participant resampling iterations, calculated separately for the lipidomic, metabolomic, and proteomic blocks. Higher values indicate greater overlap and therefore greater feature-set stability.

Repeated grouped five-fold cross-validation was used for the Pfizer-BioNTech and Moderna comparisons, whereas leave-one-participant-out cross-validation was used for AstraZeneca. AstraZeneca validation results should be interpreted cautiously because only seven participants were available. Overall, classification performance was highly reproducible, whereas the precise composition of the selected molecular feature sets showed low-to-moderate stability across resampling.

**Supplementary Figure S27: Integrated multi-omics analysis of the Pfizer-BioNTech vaccine response.** (**A–C**) DIABLO loading plots highlighting the top discriminant features contributing to separation of post-vaccination versus baseline samples across (**A**) lipids, (**B**) metabolites, and (**C**) proteins. Positive loadings indicate post-vaccination enrichment, while negative loadings represent baseline-associated features. (**D**) Circos plot illustrating cross-omics associations (|r| > 0.7) between discriminant lipid, metabolite, and protein features. Red lines denote positive correlations; blue lines denote negative correlations.

**Supplementary Figure S28: Integrated multi-omics analysis of the Moderna vaccine response.** (**A–C**) DIABLO loading plots highlighting the top discriminant features contributing to separation of post-vaccination versus baseline samples across (**A**) lipids, (**B**) metabolites, and (**C**) proteins. Positive loadings indicate post-vaccination enrichment, while negative loadings represent baseline-associated features. (**D**) Circos plot illustrating cross-omics associations (|r| > 0.7) between discriminant lipid, metabolite, and protein features. Red lines denote positive correlations; blue lines denote negative correlations.

**Supplementary Figure S29: Integrated multi-omics analysis of the AstraZeneca vaccine response.** (**A–C**) DIABLO loading plots highlighting the top discriminant features contributing to separation of post-vaccination versus baseline samples across (**A**) proteins, (**B**) lipids and (**C**) metabolites. Positive loadings indicate post-vaccination enrichment, while negative loadings represent baseline-associated features. (**D**) Circos plot illustrating cross-omics associations (|r| > 0.7) between discriminant lipid, metabolite, and protein features. Red lines denote positive correlations; blue lines denote negative correlations. **(E)** Hierarchical clustering heatmap of discriminative feature across timepoints.
